# Expanding a peptide-covalent probe hybrid for PET imaging of *S. aureus* driven focal infections

**DOI:** 10.1186/s41181-024-00252-4

**Published:** 2024-03-26

**Authors:** Jyotsna Bhatt Mitra, Saurav Chatterjee, Anuj Kumar, Elina Khatoon, Ashok Chandak, Sutapa Rakshit, Anupam Bandyopadhyay, Archana Mukherjee

**Affiliations:** 1 Radiopharmaceuticals Division, Bhabha Atomic Research Centre (BARC), Mumbai, India; 2 Biomimetic Peptide Engineering Laboratory, Department of Chemistry, Indian Institute of Technology Ropar, Ropar, Punjab India; 3 Board of Radiation and Isotope Technology, Navi Mumbai, India; 4 Radiation Medicine Centre, Parel Mumbai, India; 5 Homi Bhabha National Institute, Anushaktinagar, Mumbai, India

**Keywords:** Antimicrobial peptides, Ubiquicidin, Infection imaging, ^68^Ga radiopharmaceuticals, Covalent probe

## Abstract

**Background:**

The urgent demand for innovative theranostic strategies to combat bacterial resistance to antibiotics is evident, with substantial implications for global health. Rapid diagnosis of life-threatening infections can expedite treatment, improving patient outcomes. Leveraging diagnostic modalities i.e., positron emission tomography (PET) and single photon emission computed tomography (SPECT) for detecting focal infections has yielded promising results. Augmenting the sensitivity of current PET and SPECT tracers could enable effective imaging of pathogenic bacteria, including drug-resistant strains.UBI (29–41), an antimicrobial peptide (AMP) fragment recognizes the *S. aureus* membrane through electrostatic binding. Radiolabeled UBI (29–41) is a promising SPECT and PET-based tracer for detecting focal infections. 2-APBA (2-acetyl-phenyl-boronic acid), a non-natural amino acid, specifically targets lysyl-phosphatidyl-glycerol (lysyl-PG) on the *S. aureus* membranes, particularly in AMP-resistant strains. We propose that combining UBI with 2-APBA could enhance the diagnostic potential of radiolabeled UBI.

**Results:**

Present work aimed to compare the diagnostic potential of two radiolabeled peptides, namely UBI (29–41) and 2-APBA modified UBI (29–41), referred to as UBI and UBI-APBA. APBA modification imparted antibacterial activity to the initially non-bactericidal UBI against *S. aureus* by inducing a loss of membrane potential. The antibacterial activity demonstrated by UBI-APBA can be ascribed to the synergistic interaction of both UBI and UBI-APBA on the bacterial membrane. To enable PET imaging, we attached the chelator 1,4,7-triazacyclononane 1-glutaric acid 4,7-acetic acid (NODAGA) to the peptides for complexation with the positron emitter Gallium-68 (^68^Ga). Both NODAGA conjugates were radiolabeled with ^68^Ga with high radiochemical purity. The resultant ^68^Ga complexes were stable in phosphate-buffered saline and human serum. Uptake of these complexes was observed in *S. aureus* but not in mice splenocytes, indicating the selective nature of their interaction. Additionally, the APBA conjugate exhibited superior uptake in *S. aureus* while preserving the selectivity of the parent peptide. Furthermore, [^68^Ga]Ga-UBI-APBA demonstrated accumulation at the site of infection in rats, with an improved target-to-non-target ratio, as evidenced by ex-vivo biodistribution and PET imaging.

**Conclusions:**

Our findings suggest that linking UBI, as well as AMPs in general, with APBA shows promise as a strategy to augment the theranostic potential of these molecules.

**Supplementary Information:**

The online version contains supplementary material available at 10.1186/s41181-024-00252-4.

## Background

Bacterial infections pose serious threat to humans as antimicrobial resistance (AMR) against standard drugs often leads to millions of deaths worldwide. Recently, World Health Organization (WHO) has declared AMR as a challenge to human health and a systematic analysis estimated 4.95 million deaths related to bacterial AMR in 2019 (Antimicrobial Resistance Collaborators 2022; Walsh et al. 2023). To curb the spread of AMR there is an urgent need for minimizing the prescription of unnecessary antibiotics. Therefore, innovative diagnostic agents, compatible with current imaging modalities, are desired for developing precision medicine to fight against pathogenic bacterial infections (Zhuang et al. 2022). In recent years, significant research work on the development of non-invasive techniques for imaging bacterial infections has been reported in preclinical as well as clinical settings (Van Oosten et al. 2015; Polvoyet al. 2020; Ebenhan et al. 2018). While structural imaging techniques such as magnetic resonance imaging (MRI) and computed tomography (CT) provide excellent structural resolution for detecting advanced infections, the identification of infectious foci at an early stage remains unattainable through these methods. This limitation arises because they solely rely on late-occurring anatomic changes in disease pathophysiology, such as the host immune response and tissue damage (Kumar et al. 2008; Polvoyet et al. 2020). Functional imaging modalities, single photon emission computed tomography (SPECT), and positron emission tomography (PET), along with CT and MRI play a significant role in presenting disease status at an early stage (Polvoyet et al. 2020).

An ideal radiotracer for facilitating the expeditious diagnosis of focal infection should selectively accumulate in infection foci, be non-toxic, cost-effective, capable of distinguishing sterile inflammation from infection, and suitable for immunocompromised patients (Salmanoglu et al. 2018). The urgent need for such an ideal agent in clinical diagnosis persists, but it is currently unavailable, except for some success with SPECT and PET-based tracers (Salmanoglu et al. 2018; de Murphy et al. 2010; Bhatt et al. 2018; Mukherjee et al. 2018 ; Ebenhan et al. 2018).

An antimicrobial peptide (AMP) serving as a targeting vector for formulating an imaging agent is an attractive strategy for the specific detection of bacterial infections. Multiple groups have shown that fragments derived from Ubiquicidin originally isolated from mouse macrophages serve as a promising candiidate for PET and SPECT imaging (Hiemstra et al. 1999; de Murphy et al. 2010; Bhatt et al. 2018; Mukherjee et al. 2018; Ebenhan et al. 2018). Multiple studies have demonstrated that ^99m^Tc labeled UBI (29–41) can successfully differentiate between bacterial infection and sterile inflammation (de Murphy et al. 2010; Welling et al. 2000). Further, a recent systematic review examining the progress of ubiquicidin-based PET imaging agents provided strong case for the use of [^68^Ga]Ga-UBI as a PET based infection imaging agent (Marjanovic-Painter et al. 2023).

Strategies such as stapling antimicrobial peptides, incorporating non-natural amino acids, and employing a covalent probe, are actively pursued to develop a new generation of imaging agents and antibacterials for clinical translation (Molchanova et al. 2017; Migoń et al. 2018). It has been reported that the inclusion of a covalent probe, namely 2-acetylphenylboronic acid (2-APBA), in a peptide sequence as a modified cysteine residue aids in the selective imaging of bacterial infections, with an improved signal-to-noise ratio, through a synergistic binding approach (Aweda et al. 2019; Mitra et al. 2022). Our previous work also demonstrated that the conjugation of 2-APBA improved the salt tolerance of UBI (29–41) labeled with ^99m^Tc, possibly by eliminating the dependence on electrostatic interactions for bacterial uptake (Mitra et al. 2022).

The covalent probe 2-APBA forms an iminoboronate linkage with the amine-presenting bacterial phospholipid, lysyl-phosphatidyl-glycerol (Lysyl-PG) (Bandyopadhyay et al. 2015). Importantly, lysylation of phosphatidyl-glycerol (PG) is a widely recognized lipid mutation strategy employed by *S. aureus* to evade the binding of cationic AMPs (Roy 2009). Therefore, conjugating antimicrobial peptides with APBA can potentially facilitate the detection of AMP-resistant bacteria.

The bifunctional chelator 1,4,7-triazacyclononane-1,4,7-triacetic acid (NOTA) and its derivative 1, 4, 7 triazacyclononane 1-glutaric acid 4–7 acetic acid (NODAGA) form thermodynamically stable complexes with ^68^Ga (Kumar et al. 2008). Moreover, these macrocyclic chelators contribute to forming hydrophilic ^68^Ga-complexes facilitating their faster renal clearance, ultimately resulting in improved target to non-target ratios. Hence, UBI fragments conjugated to macrocyclic chelator NODAGA were used in this study for their evaluation as potential infection imaging probes (Kumar et al. 2008). Since 2-APBA is attached to peptide as an acetyl-cysteine conjugate (AcCys-2-APBA), the latter was used as a control in the present study.

**Fig. 1:**
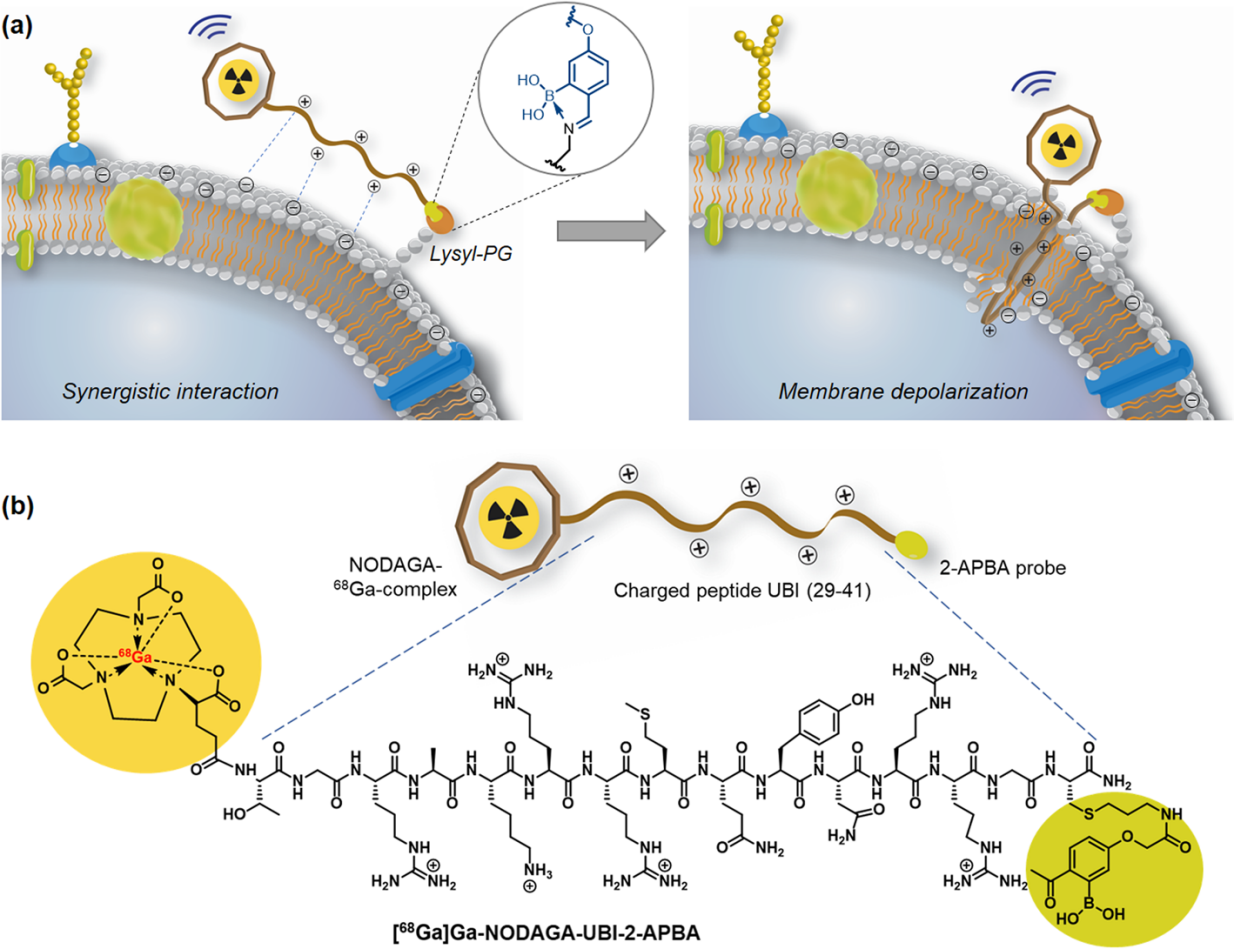
**a** Synergistic interaction of UBI-APBA for bacterial detection and membrane depolarization.** b** Chemical structure of [^68^Ga] Ga-UBI-APBA formulation

Herein, we report the potency of 2-APBA incorporated UBI (29–41) on *S. aureus* growth and survival, including its mechanism of action (Fig. 1a). For convenient ^68^Ga labeling, we attached peptides to the bifunctional chelator NODAGA (Fig. 1b). The resulting Ga-68 complexes were examined both in vitro and in vivo to assess the potential of ^68^Ga-labeled UBI derivatives for detecting bacterial infections. We observed improved bacterial uptake of the APBA conjugate potentially through synergy between electrostatic and covalent interaction while recognizing the bacterial membrane. A tighter binding between UBI-APBA and bacterial membrane can lead to cell death via processes such as membrane depolarization (Fig. 1a). Our preliminary findings indicate that the modification of AMPs and AMP fragments such as UBI with 2-APBA holds promise as a strategy to improve their specific uptake by bacteria, potentially leading to enhanced diagnostic outcomes.

## Results

### Peptide synthesis

**Fig. 2:**
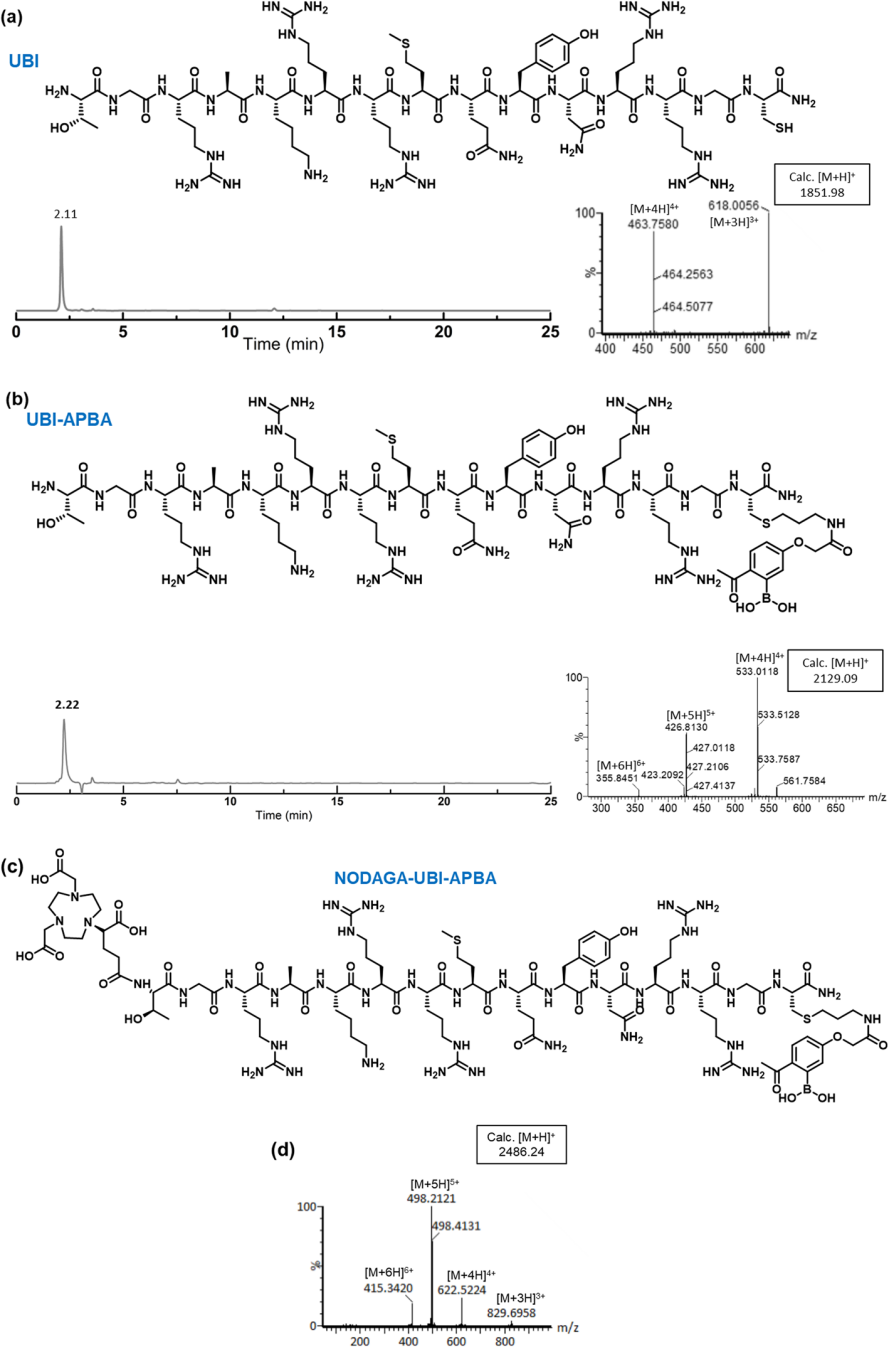
**a** UBI peptide data: HPLC purity at 220 nm and mass datum. **b** UBI-APBA peptide data: HPLC chromatogram at 220 nm showing > 95% purity of the product and mass datum confirms the product’s identity. **c**, **d** NODAGA-UBI-APBA data: its chemical structure, and mass datum confirms the product’s identity

The peptide synthesis was performed using the conventional Fmoc-based solid-phase peptide synthesis method. Subsequently, the 2-APBA moiety was affixed to a programmed cysteine residue located at the C-terminus using a thiol-ene click reaction. To facilitate this attachment, an alkene handle was strategically positioned alongside the 2-APBA moiety in accordance with the procedure outlined (Chatterjee et al. 2023). Further, NODAGA (^t^Bu)_3_ was coupled at the N-terminus. Structures and mass data (ESI-MS) of the UBI peptide and conjugates (UBI-APBA and NODAGA-UBI-APBA) are presented in Fig. 2. The product was obtained in high conversion yields (Additional file 1). The mass and purity of the peptide and conjugates were determined by using LC-MS (Waters, USA) and HPLC (Shimadzu, Japan), respectively.

### Effect of peptide-covalent probe hybrid on the growth and survival of *S. aureus*

**Fig. 3:**
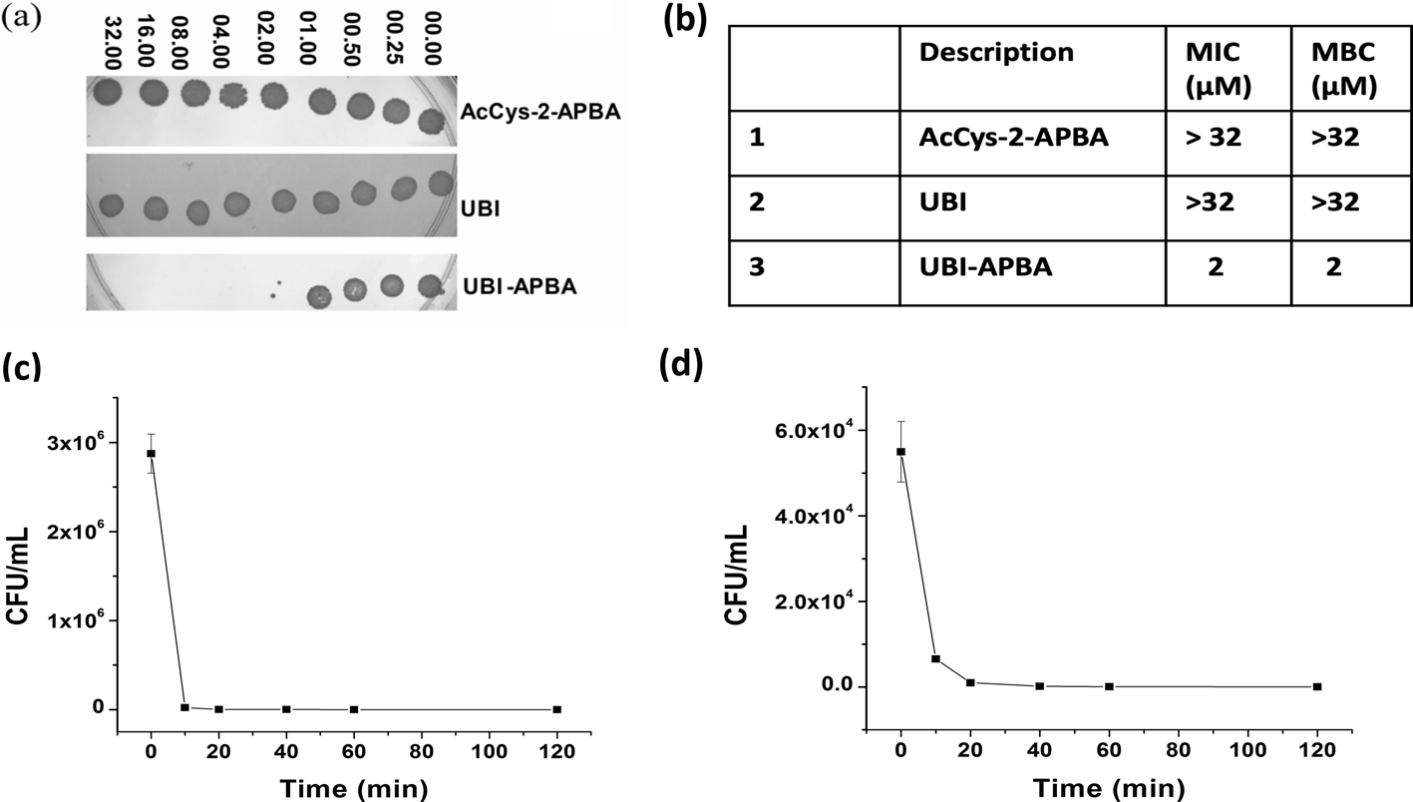
**a**, **b** Bactericidal impact of AcCys-2-APBA, UBI and UBI-APBA conjugate on *S. aureus*. **b** Tabulated MIC and MBC values. **c** CFU/mL versus time in minutes (min) graph showing the kinetics of killing by UBI-APBA at MIC with *S. aureus* **d** and its small colony variants (SCV)i

Minimum inhibitory concentrations (MICs) for AcCys-2-APBA, UBI and UBI-APBA conjugate were determined in a microtiter plate format by resazurin (alamar blue) reduction assay. The MICs were determined as the lowest concentration at which the conversion of the resazurin dye from blue to pink was absent. These values for all the compounds were determined through visual observation and are tabulated in Fig. 3b. Conversely, the minimum bactericidal concentrations (MBC) were determined using the spot assay shown in Fig. 3a, where the absence of a bacterial lawn indicated the minimum required concentration for bacterial killing. Both MIC and MBC were found to be 2 µM for UBI-APBA indicating that it had growth inhibitory (bacteriostatic) as well as bactericidal effect on *S. aureus* (Fig. 3a, b). However, AcCys-2-APBA as well as UBI had no such effect on the growth of *S. aureus.* Although UBI has been previously reported as bactericidal against *S. aureus*, the experimental conditions and strain utilized were different (Brouwer et al. 2006). Nevertheless, our previous mechanistic studies show that selective recognition of bacterial membrane was possible by UBI (Bhatt Mitra et al. 2020).

UBI-APBA demonstrated a time-dependent killing effect on *S. aureus* cells (Fig. 3c). Notably, the APBA conjugate of UBI also eradicated small colony variants (SCV) of *S. aureus* (Fig. 3d). The adoption of the SCV phenotype under environmental stress is a recognized survival mechanism for *S. aureus * (Loss et al. 2019). In summary, the conjugation of 2-APBA to UBI imparted therapeutic potential to the peptide.

### Toxicity studies

**Fig. 4:**
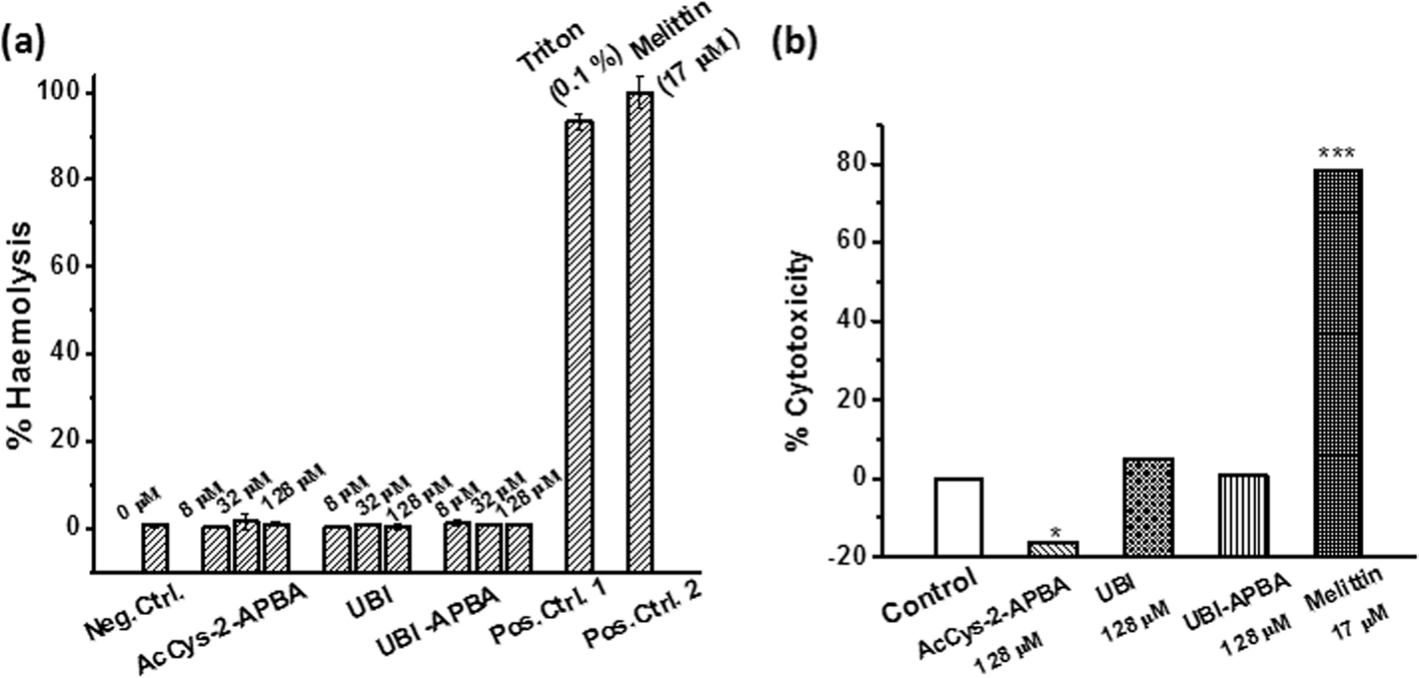
Toxicity of AcCys-2-APBA, UBI and UBI-APBA conjugate against human erythrocytes and HEK 293 cells. **a**. Bar graphs showing % haemolysis caused by indicated concentrations of AcCys-2-APBA, UBI and UBI-APBA. Untreated erythrocytes were used as negative control (Neg. Ctrl.), whereas Triton-X-100 (0.1%) and melittin (17 µM) treated cells were used as positive controls (Pos. Ctrl.) **b**. Cell cytotoxicity of UBI and APBA conjugate toward HEK 293 cells *indicates *p* < 0.05, whereas ***indicates *p* < 0.0005 (*n* = 3)

The haemolysis assay is widely used to assess the therapeutic potential of a given AMP. AMPs that cause ruptured RBCs at their MICs naturally have low therapeutic potential. To evaluate whether UBI-derived peptides were toxic to human RBCs, a haemolysis assay was conducted at concentrations higher than the observed MIC (2 µM) for UBI-APBA, with appropriate controls. It was observed that at tested concentrations, all three compounds exhibited no haemolysis while melittin and 0.1% (v/v) triton X-100 (positive controls) induced haemolysis in erythrocytes (Fig. 4a). The cell cytotoxicity assay demonstrated that the treatment of HEK 293 (immortalized non-cancer cells) with UBI and UBI-APBA induced minimal cytotoxicity, similar to the untreated control (Fig. 4b). Additionally, melittin (positive control) was found to be cytotoxic to HEK 293 cells (Fig. 4b). However, AcCys-2-APBA appeared to induce significant proliferation in the cells. This observation can be explained by the fact that AcCys-2-APBA is an amino acid conjugate. Therefore, it is possible that the cells utilize it as a nitrogen source, resulting in enhanced proliferation when used alone.

In summary, the haemolysis data, together with the cytotoxicity assay, indicates that AcCys-2-APBA, UBI, and UBI-APBA were neither hemolytic nor cytotoxic to mammalian cells at tested concentrations.

### Mechanism of bacterial killing by UBI-APBA

**Fig. 5:**
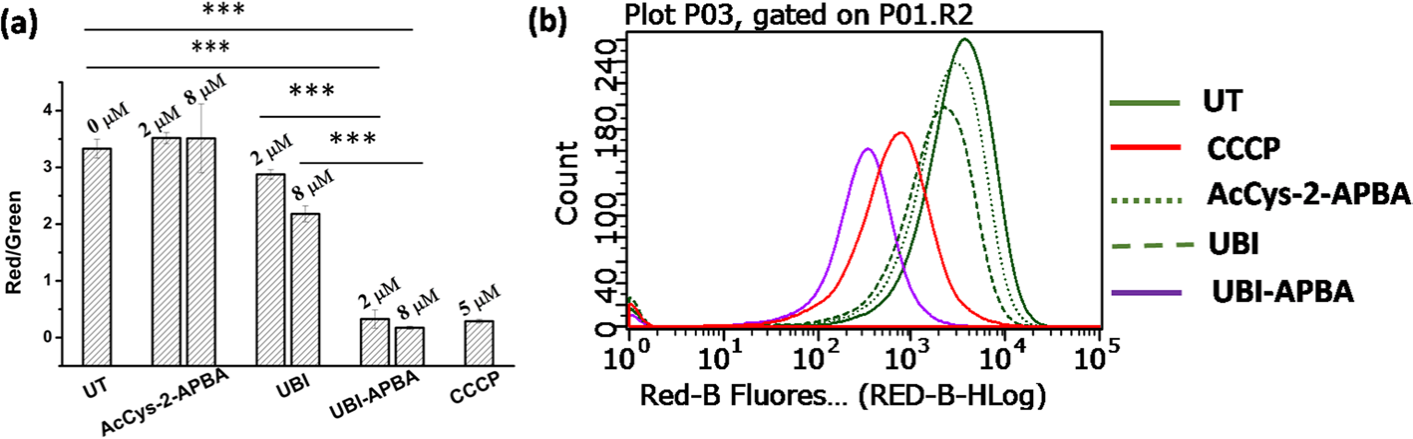
**a** Bar graph depicting the ratio of red and green fluorescence intensities of bacterial samples with designated treatments (UT- untreated). ***indicates *p* < 0.0005 (*n* = 3). **b** Histogram showing live (high red) and depolarized *S. aureus* (low red) for untreated, CCCP (positive control), AcCys-2-APBA, UBI and UBI-APBA treated cells respectively at 8 µM (4 × MIC of UBI-APBA)

**Fig. 6:**
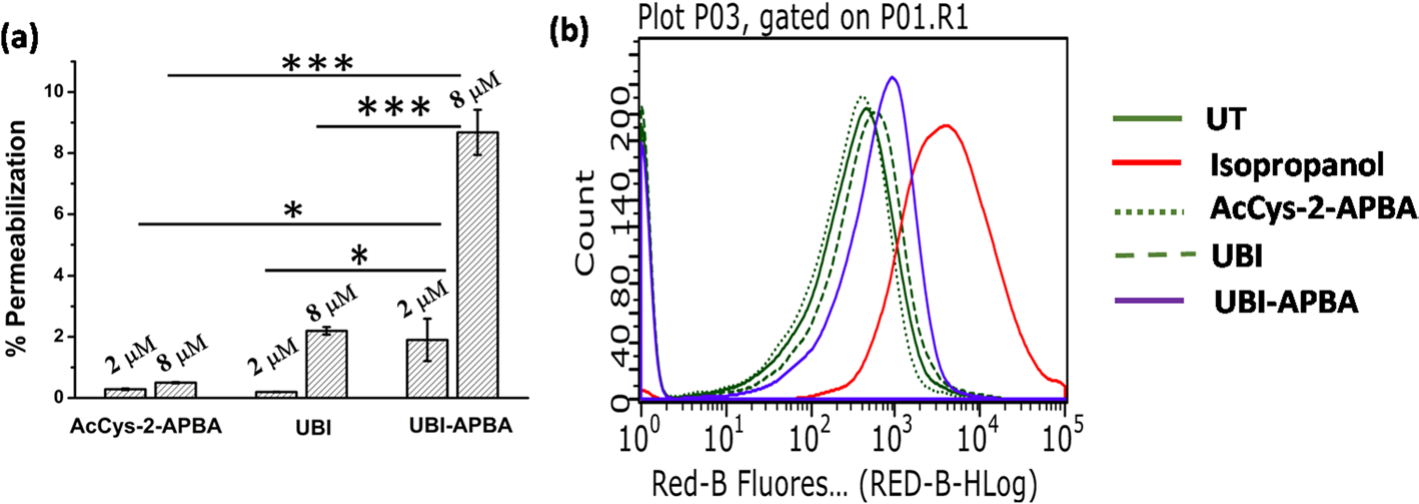
**a** Bar graph showing normalized permeabilization of samples at MIC and 4 × MIC of UBI-APBA. *indicates *p* < 0.05, whereas ***indicates *p* < 0.0005 (*n* = 3). **b** Histogram showing PI uptake in *S. aureus* for untreated (UT), Isopropanol (positive control), AcCys-2-APBA, UBI and UBI-APBA treated cells, respectively at 8 µM (4 × MIC of UBI-APBA

AMPs are reported to destroy bacterial cells by permeabilizing the membrane, followed by depolarization (Mitra et al. 2020; Bhatt Mitra et al. 2020). To explore if UBI-APBA utilized this strategy to adversely affect the survival of *S. aureus*, depolarization and permeabilization of *S. aureus* membrane was studied by flow cytometry. It was found that as opposed to AcCys-2-APBA and UBI, UBI-APBA severely depolarized bacterial membrane characterized by a low red to green fluorescence intensity ratio like CCCP (Carbonyl cyanide m-chlorophenyl hydrazone) control at both MIC and 4 × MIC (Fig. 5). Furthermore, we investigated the permeabilization of the *S. aureus* membrane by assessing propidium iodide uptake (PI). As PI is a red fluorescent dye selectively taken up by cells with compromised membrane integrity, our results revealed that, in comparison to the isopropanol control, UBI-APBA induced minimal permeabilization of the *S. aureus* membrane (Fig. 6). Conversely, AcCys-2-APBA and UBI did not show any effect on *S. aureus* membrane permeability. Nevertheless, the minimal permeabilization caused by UBI-APBA seems sufficient to depolarize *S. aureus* membrane.

### Radiolabeling and stability studies

**Fig. 7:**
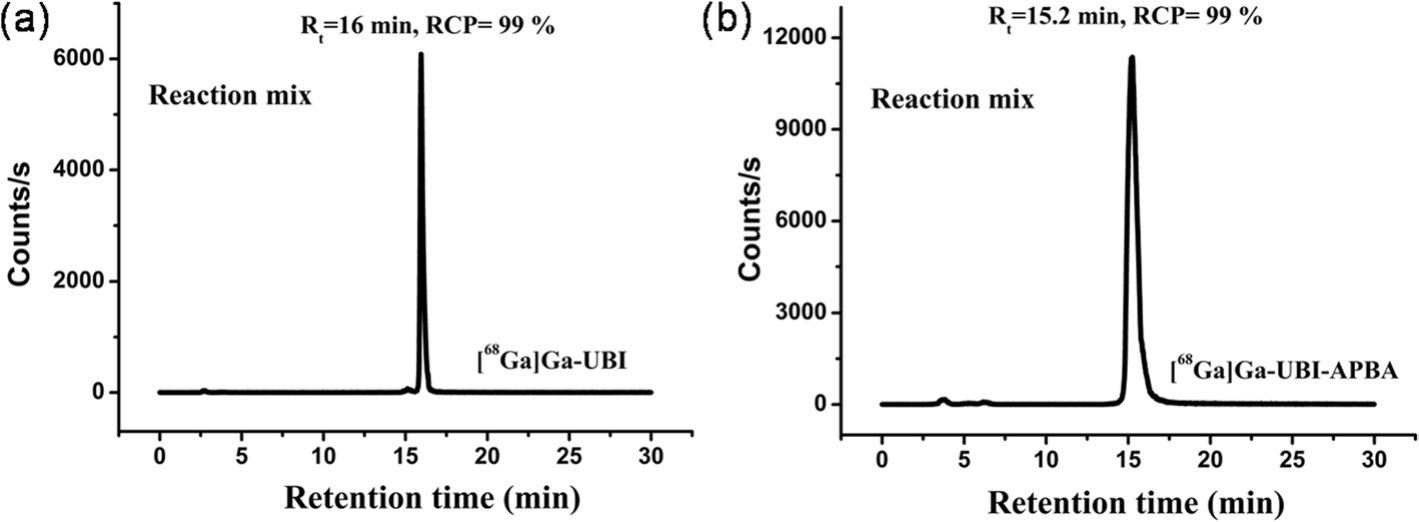
HPLC radiochromatograms showing RCP and retention times of **a** [^68^Ga]Ga-UBI **b** [^68^Ga]Ga-UBI-APBA

**Fig. 8:**
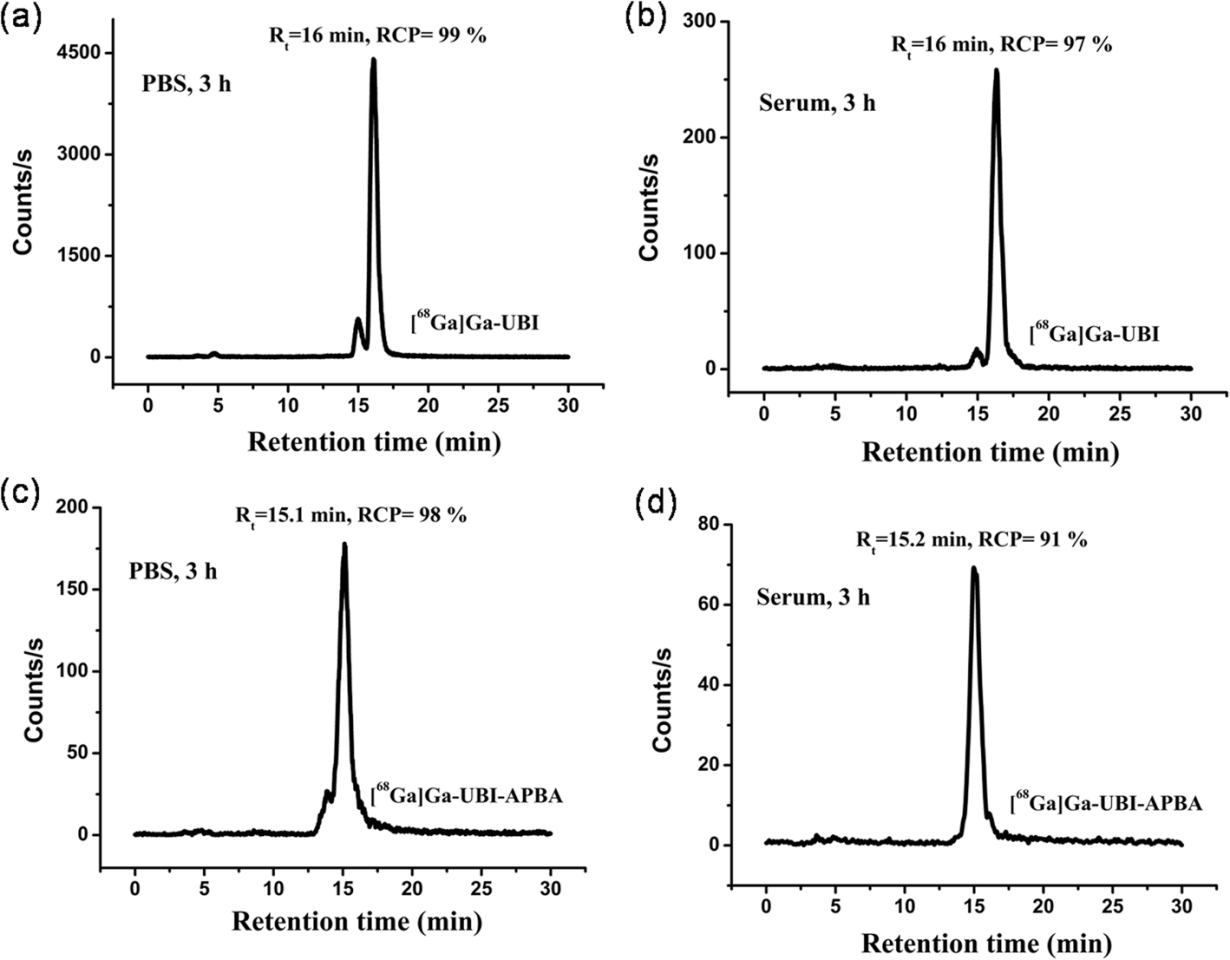
HPLC radiochromatograms showing in vitro stability in PBS at 3 h **a** [^68^Ga]Ga-UBI **c** [^68^Ga]Ga-UBI-APBA. **b** and **d** depict their stabilities in serum (3 h) respectively

The optimized protocol for preparing [^68^Ga]Ga-UBI complexes resulted in consistently superior radiochemical purity (RCP > 99%, *n* = 4). Radiochromatograms of the [^68^Ga]Ga-UBI and [^68^Ga]Ga-UBI-APBA complexes immediately after formulation are shown in Fig. 7. All the complexes were stable in PBS as well as in serum till 3 h, as shown in Fig. 8. This was evident from the absence of peak corresponding to free ^68^Ga, which shows a retention time of 3.1 ± 0.03 min. Partition coefficients or Log P values of -3.8 ± 0.15 (*n* = 4), and − 3.7 ± 0.13 (*n* = 4) were observed for [^68^Ga]Ga-UBI and [^68^Ga]Ga-UBI-APBA, respectively. These negative log P values revealed that [^68^Ga]Ga-UBI complexes are hydrophilic.in nature. The hydrophilicity of complexes is a predictor of fast renal clearance, which is desirable for diagnostic radiopharmaceuticals.

### In vitro uptake assay

**Fig. 9:**
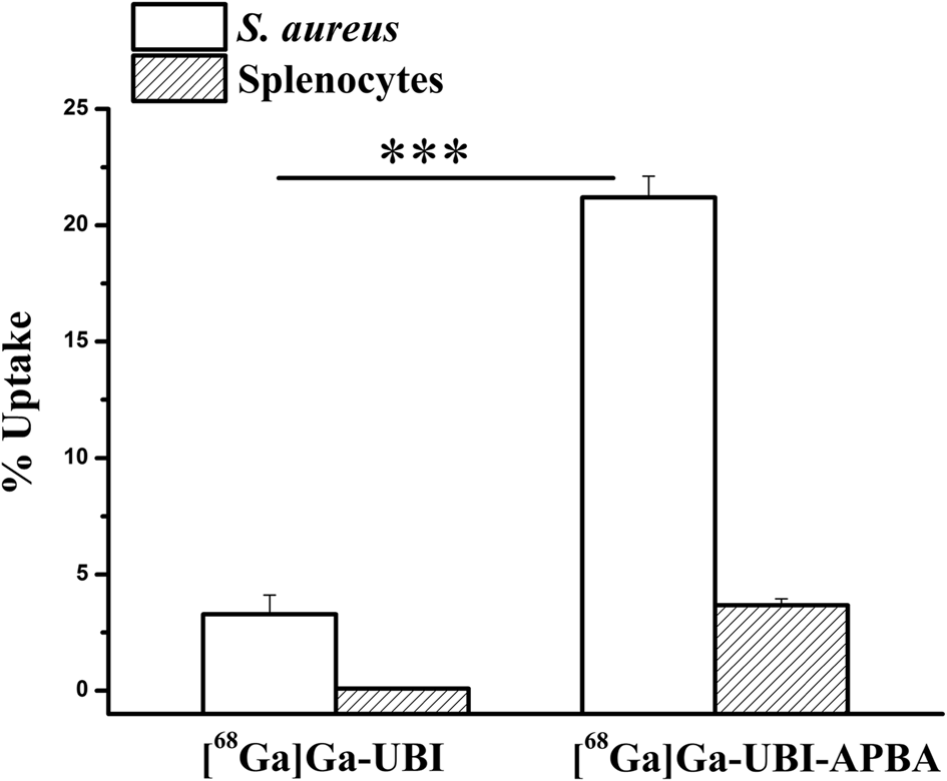
Uptake of [^68^Ga]Ga-UBI and [^68^Ga]Ga-UBI-APBA in *S. aureus* and splenocytes. ***indicates *p* < 0.0005 (*n* = 3)

For determining the selectivity of the radiolabeled complexes towards bacterial cells, an in vitro uptake assay was performed in *S. aureus* as well as mice splenocytes representing host immune cells. Uptake of [^68^Ga]Ga-UBI complexes was observed in *S. aureus*, as depicted in Fig. 9. It was found that [^68^Ga]Ga-UBI-APBA showed significantly higher uptake compared to the unconjugated peptide in *S. aureus* cells. This can be explained by the stronger covalent interaction (iminoboronate linkage) between the 2-APBA group and bacterial phospholipid Lysyl-PG, working in synergy with the comparatively weaker electrostatic interaction between the positively charged amino acids of UBI and bacterial phospholipids. Conversely, [^68^Ga]Ga-UBI complexes showed minimal uptake in splenocytes, indicating the selectivity of the complexes towards *S. aureus*. Selectivity of UBI derivatives has been previously established through biophysical experiments (Bhatt Mitra et al. 2020). Our toxicity data with human erythrocytes and HEK 293 cells also confirmed the selectivity of UBI and its 2-APBA conjugate towards the *S. aureus* cells (Fig. 4). Several AMPs that demonstrate strong antibacterial activity encounter challenges due to their toxicity to human red blood cells (RBCs), leading to a diminished therapeutic potential (Mitra et al. 2020). The low therapeutic index or TI (TI = Haemolytic dose /MIC causing 50% lysis or HD_50_) of such AMPs make their clinical translation extremely difficult. In the present study, the conjugation of 2-APBA to UBI resulted in selective activity against bacteria, as anticipated due to the specificity of UBI towards *S. aureus* membranes (Bhatt Mitra et al. 2020).

#### In vivo evaluation

**Fig. 10:**
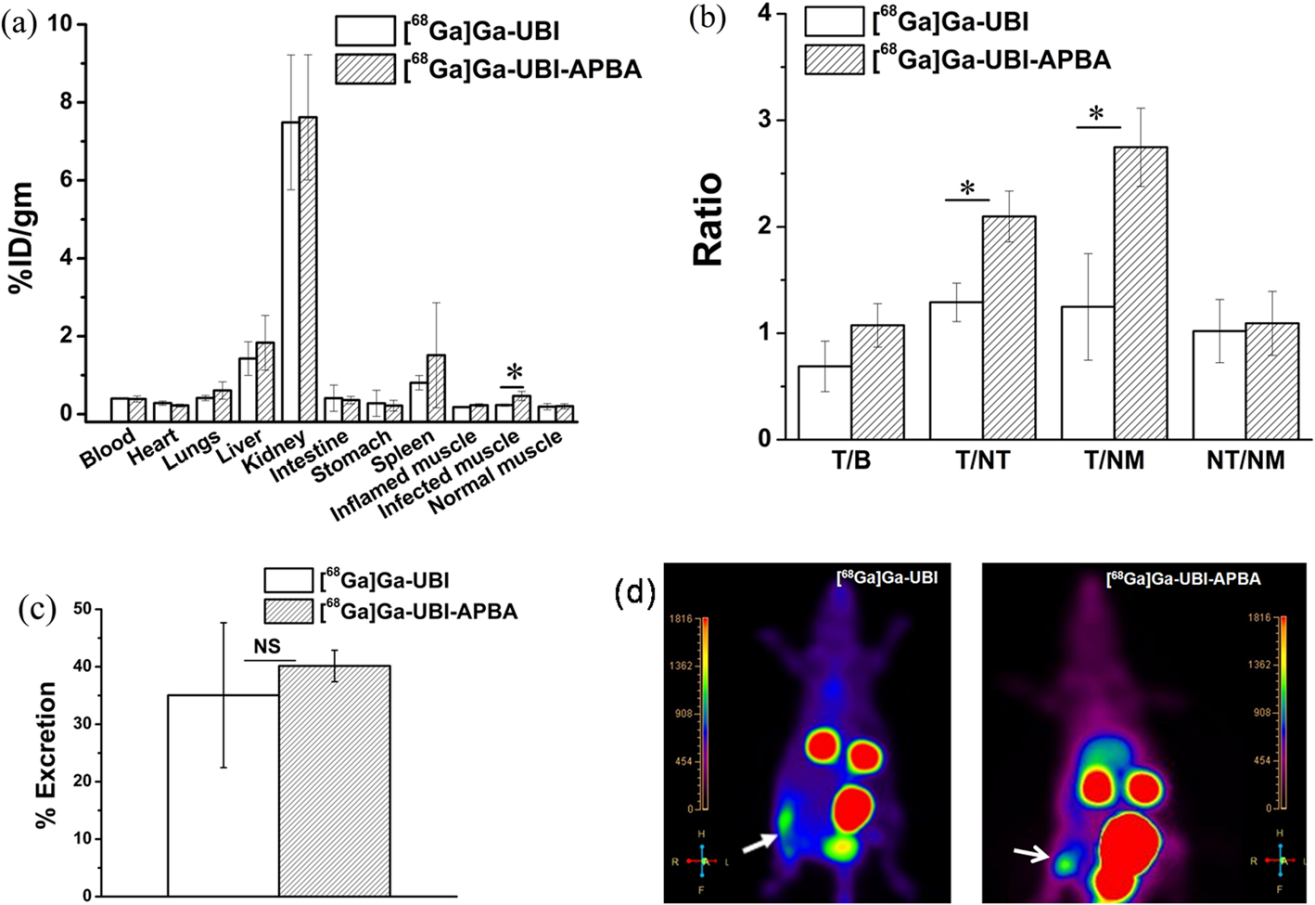
In vivo studies in rat model of *S. aureus* infection. **a** *Ex-vivo* biodistribution of [^68^Ga] Ga-UBI complexes in rat model of infection and inflammation. **b** Bar graph comparing the efficacy of in vivo detection of *S. aureus* by ^68^Ga tracers. T = % ID/gm at the target or site of infection, B = Blood, NT = non-target or site of sterile inflammation, NM = Normal muscle. **c** Bar graph showing % excreted activity at 1 h post injection (p.i.) for respective [^68^Ga]Ga-UBI complexes. **d** PET images showing accumulation of tracer at the site of infection (white arrow, right thigh muscle) in rat model of infection and no accumulation in sterile inflammation (left thigh muscle), images show renal clearance through kidneys and bladder. *Indicates a *p* value of < 0.05 (*n* = 4), NS denotes non-significant difference

The ^68^Ga complexes were assessed in rat models of infection and inflammation through *ex-vivo* biodistribution and PET imaging. Both tracers exhibited the highest accumulation in kidneys, liver, and spleen, in that order (Fig. 10a). Accumulation in kidneys and liver suggests clearance primarily through the renal route, followed by hepatobiliary pathway. A primarily renal clearance was anticipated for these ^68^Ga complexes due to their high hydrophilicity as previously mentioned.

In all other organs, the accumulation of tracers was minimal. The bar graph in Fig. 10b illustrates the accumulation of tracers at the site of infection (T = Target) in relation to blood (B), sterile inflammation (NT = Non-target), and normal muscle (NM). It is evident that [^68^Ga]Ga-UBI-APBA could distinguish the target site significantly better compared to [^68^Ga]Ga-UBI, as reflected in the improved T/NT ratio (Fig. 10b). Figure 10c shows that up to 40% of the activity was cleared from the rats within 1 h of injection, primarily through the renal route. To summarize, *ex-vivo* biodistribution data established [^68^Ga]Ga-UBI-APBA as a superior tracer for imaging *S. aureus* driven focal infection as compared to [^68^Ga]Ga-UBI.

A bacterial recovery experiment was conducted at the end of the biodistribution, revealing that approximately 3 × 10^8^ CFU were recovered from the infection foci in the rats. In contrast, the bacterial recovery from sterile inflammation was insignificant, with only ~ 179 CFUs detected. Notably, no *S. aureus* colonies were detectable in the blood indicating focal nature of the infection.

The PET images of rats injected with [^68^Ga]Ga-UBI tracers show that both the tracers could identify the target site as well as distinguish it from the site of sterile inflammation (Fig. 10d). This observation corroborated the biodistribution data. Moreover, clearance of activity largely through renal route was observed, as evident from uptake in the kidneys and urinary bladder.

## Conclusion

Radiolabeled UBI derivatives have shown promising results for in situ detection of *S. aureus* driven infection by SPECT and PET imaging techniques. We observed that [^68^Ga] Ga-UBI-APBA showed significantly higher uptake compared to its unconjugated counterpart in *S. aureus* cells. This result resembles the findings obtained from the study conducted using ^99m^Tc-labeled UBI-APBA (Mitra et al. 2022). Uptake studies in mouse splenocytes, combined with our toxicity data in human erythrocytes and HEK 293 cells, suggest that 2-APBA conjugation did not compromise the selectivity of UBI towards *S. aureus* cells. Furthermore, our in vitro and in vivo studies in animal models demonstrated that [^68^Ga]Ga-UBI-APBA had significantly better uptake compared to [^68^Ga]Ga-UBI in *S. aureus*. Therefore, our preliminary data confirm the potential of UBI-APBA peptide-covalent probe hybrid for diagnosing bacterial infections.

Our study also showed that the conjugation of the covalent probe, 2-APBA, to UBI drastically impacted the growth and survival of *S. aureus* by depolarization of the bacterial membrane. Hence, we propose that the conjugation of 2-APBA could serve as an effective method to enhance the antibacterial activity of existing AMPs without introducing toxicity against host cells.

### Methods

#### Peptide synthesis

Peptide synthesis was carried out using the conventional Fmoc chemistry on the solid-phase. The AcCys-2-APBA-alkene covalent probe was installed on the prearranged cysteine moiety at C-terminus via thiol-ene click reaction as per the reported procedure (Additional file 1; Mitra et al. 2022). Similarly, NODAGA-UBI-APBA was synthesized by introducing NODAGA chelator at the N-terminus of UBI as shown in Fig. 2. All peptides were purified through HPLC, structural identity was confirmed via ESI-HRMS, and were subjected for further studies.

### Determination of minimum inhibitory concentration and bactericidal concentration of UBI derivatives

A single colony of *S. aureus*, ATCC 25,923 (Microbiologics) was inoculated in Mueller Hinton broth (MHB) and incubated overnight at 120 rpm (revolutions per minute) and 37 °C. The experiment was carried out in a 96-microtiter plate using a broth dilution assay wherein all the peptides (ABI scientific) were diluted two-fold in each step-in media (M9 supplemented with 0.01% Tryptone). Cells were washed twice with media and diluted to a final concentration of 10^5^ colony forming units (CFU) at each well. The final volume in each well was 200 µL. 5 µL aliquots were pipetted out from wells, spotted onto MHB-agar plates, and incubated overnight at 37 °C to perform the spot assay for determination of minimum bactericidal concentration (MBC). After 24 h, 30 µL of 0.75 mg/mL of resazurin dye (HiMedia Labs) was added in each of the wells and kept for incubation overnight at 37 °C with mild shaking. The next day, the 96 well plates as well as MHB-agar plates, were observed, and results were recorded. The experiment was carried out in triplicates. As mentioned earlier, *S. aureus* was cultured and washed with media to carry out a time-kill assay. Experiments were performed in microfuge tubes. ~10^6^ cells were incubated with minimum inhibitory concentration (MIC) of UBI-APBA (2 µM) at a final volume of 500 µL adjusted with M9 media. Cells and UBI were incubated in media at 37 °C at 120 rpm. 50 µL aliquots from each reaction mixture were removed at required time intervals. These aliquots were plated on the MHB agar plates and incubated at 37 °C. The following day, colonies were counted to get CFU. Surviving fractions were plotted against time. Origin graphing and analysis 6.0 Pro software was used for plotting and analysis of data.

### Haemolysis assay

Fresh human blood samples were obtained from healthy donors with the approval of the institutional medical ethics committee. Erythrocytes were isolated from heparinized human blood by centrifugation at 2000*g* for 5 min after washing three times with PBS at 4 °C. After removing the supernatant, the cell pellet was resuspended in 2 mL of PBS, and cells were diluted at 4% (v/v) for the haemolysis experiment. Erythrocyte suspension was incubated with various concentrations of peptides (4 × MIC = 8 µM, 16 × MIC = 32 µM, 64 × MIC = 128 µM) for 30 min at 37 °C at a final volume of 200 µL in microfuge tubes in duplicates. Samples were centrifuged at 2000*g* for 5 min, and the absorbance of supernatants was measured at 540 nm using a polarstar omega plate reader from BMG Labtech. Erythrocytes lysed with 0.1% triton X-100 and 50 µg/mL melittin were used as positive controls. Percentage haemolysis was calculated using the formula: Haemolysis (%) = (A_SAMPLE_ − A_NG_)/(A_PC_ − A_NG_) × 100[A_SAMPLE_ = Absorbance recorded for samples at 540 nm, A_NG_ = Absorbance recorded for untreated (negative control) erythrocytes at 540 nm, A_PC =_ Absorbance recorded for Melittin treated (positive control) erythrocytes at 540 nm]. One-way ANOVA was used to establish the presence of significant groups, and Tukey’s test was subsequently utilized to conduct the comparison of means.

### Cell cytotoxicity assay

Human embryonic kidney (HEK 293) cells were maintained in DMEM supplemented with 10% FBS and 1% antibiotic-antimycotic solution at 37 °C, 5% CO_2_ in humidified atmosphere.

Cell cytotoxicity was measured by performing 3-(4,5-dimethylthiazol-2-yl)-2,5-diphenyltetrazolium bromide) (MTT) assay. Briefly, 2000 cells/100 µL were seeded in a 96-well plate and kept overnight in incubator at 37 °C, 5% CO_2_. 10 µL of drugs (AcCys-2-APBA, UBI, UBI-APBA) at a concentration of 128 µM (64 × MIC of UBI-APBA) was added to the wells and incubated for 24 h. Melittin (17 µM) was used as a positive control for cytotoxicity assay. Following incubation, 10 µL of MTT was added to each well and after 3 h the supernatant was removed and 100 µL of dimethyl sulfoxide (DMSO) was added. After 1 h incubation, the absorbance was recorded at 570 nm. One-way ANOVA was used to establish the presence of significant groups, and Tukey’s test was subsequently utilized to conduct the comparison of means.

### Flow cytometry

10^7^ CFU/mL log phase bacteria (*S. aureus*) were washed with media and incubated with compounds at MIC and 4 × MIC of UBI-APBA (8 µM) for 1 h at 37 °C and 120 rpm. For the permeabilization study, peptide-treated cells were washed with saline followed by staining with DNA stain, propidium iodide (PI) at a final concentration of 45 µM (30 min at 37 °C and 120 rpm). Isopropanol treated cells were used as positive control. The % permeabilization was calculated after normalizing the values with respect to the untreated (0% permeabilization) and isopropanol treated cells (100% permeabilization).

Positive control for membrane depolarization studies was prepared by incubating bacteria under similar conditions with 5 µM CCCP. Samples were stained with membrane potential sensing dye 3,3-Diethyloxacarbocyanine, iodide (DiOC2(3) at a final concentration of 30 µM for 30 min at 37 °C and 120 rpm. Low red to green ratio comparable to CCCP control represented the depolarized cells. All samples were analyzed by Guava EasyCyte flow cytometer. Ratio of mean fluorescent intensities (MFI) for red and green was plotted for all the samples. Paired t-test was carried out to find out the significantly different groups.

### Radiolabeling of NODAGA-UBI conjugates with ^68^Ga

^68^Ga for the study was eluted from a ^68^Ge/^68^Ga generator 740 MBq (20 mCi) from ITG using 0.05 N HCl. The amount of peptide conjugate, ^68^Ga activity, sodium acetate and reaction conditions were optimized to achieve maximum complexation yield. Radiolabeling was performed by adding ^68^Ga (74 MBq) activity to ~ 20 nmoles of peptide conjugates in 0.5 M sodium acetate buffer (molar activity = 3.7 MBq/nmol). The reaction was carried out at 90 °C for 10 min at pH 3.5–4. The High-performance liquid chromatography (HPLC) was employed to determine radiochemical purity. A dual pump HPLC unit with a C-18 reversed phase HiQ-Sil (5 μm, 25 cm × 0.46 cm) column was used for this purpose. The elution was checked by following UV signal at 214 nm and a radioactivity signal using NaI (Tl) detector. Water (A) and acetonitrile (B) mixture with 0.1% trifluoroacetic acid was utilized as the mobile phase and gradient elution with the following composition: 0–2 min: 5% B, 2–32 min: 65% B, 32–35 min: 5% B was used for the separation of free ^68^Ga and ^68^Ga complexes. The flow rate was kept at 1 mL/min.

### In vitro stability studies with ^68^Ga labeled UBI complexes

To assess the stability of the ^68^Ga labeled peptide conjugates in PBS, 50 µL of complexes were added to 450 µL of PBS (1:10 dilution) and incubated at 37 °C for 3 h. Aliquots were taken at the end of incubation and analyzed by HPLC, as described previously. Similarly, to estimate the integrity of the ^68^Ga complexes in serum, incubation was done with human serum at 1:10 dilution for 3 h at 37 °C. Samples were precipitated with 2% trichloroacetic acid (TCA), separated by centrifuging at 10,000*g* for 5 min, and analyzed by HPLC.

Partition coefficients for ^68^Ga complexes were determined by mixing 25 µL of sample with 975 µL water and 1 mL octanol (octanol: water = 1:1). Thereafter, the mixture was vortexed and incubated for 5 min, followed by centrifugation for 5 min at 3000 rpm for separation of aqueous and organic phase. NaI (Tl) counter was used to measure the activity associated with both the phases and log P value [logarithm of the activity concentration in n-Octanol / activity concentration in the aqueous layer] was calculated.

### In vitro uptake assay

In-vitro uptake assay was performed as per the optimized protocol (Mitra et al. 2022) ~ 10^8^ CFU of bacteria was used for uptake and inhibition studies. Cells were washed with sodium phosphate buffer (15 mM sodium phosphate buffer, 0.01% tween 80 (v/v), 0.1% acetic acid (v/v), 150 mM NaCl, pH 5) and incubated with [^68^Ga]Ga-UBI complexes at concentration of 0.2 µM in a final volume of 1 mL in microfuge tubes. Incubation was carried out for 1 h at ∼ 6 °C with constant mixing. Inhibition studies were carried out by pre-incubation at ∼ 6 °C for 1 h with 100-fold excess of unlabeled UBI compared to tracer concentration followed by addition of tracers. Activity associated with bacteria was separated from free activity by centrifugation at 3000*g*. Cells were washed twice with incubation buffer, and activity associated with pellet was measured using NaI (Tl) scintillation counter, and % radioactivity associated with the bacterial cells was estimated. Paired t-test was utilized to estimate statistically significant differences among groups.

Further, a splenocyte uptake assay was carried out for ^68^Ga complexes. Briefly, a BALB/c mouse was sacrificed, and its spleen was removed and kept in 10 mL Dulbecco’s modified eagle medium (DMEM) containing 10% fetal calf serum (FCS). A suspension of splenocytes was prepared using a cell sieve (Sigma Aldrich, MO, USA). The suspension was centrifuged at 2000 rpm for 5 min. The pellet was dislodged, and 4.5 mL of chilled water was added to lyse red blood cells by osmotic shock. Precisely, after 10 s, the osmotic balance was restored by adding 10 × PBS. To remove debris, a short spin was given to the cell suspension. The supernatant was collected, centrifuged again at 2000 rpm for 5 min and the pellet was resuspended in PBS. Uptake assay with splenocytes was carried out in PBS at a cell density of ∼ 10^6^/mL. Incubation with ^68^Ga complexes and unlabeled UBI was carried out as per the protocol described for bacteria.

### In vivo evaluation

Animal experiments were performed as per regulations, guidelines and approval from the institutional animal ethics committee. Wistar rats injected with ∼ 5 × 10^8^ CFU of heat-killed (left thigh), and live *S. aureus* (right thigh) showed visible signs of inflammation and infection, respectively i.e., redness, swelling and difficulty in mobility. To prepare heat-killed bacteria, *S. aureus* suspended in PBS were exposed to 99 ^o^C temperature for ~ 3 h in a heating block. Effectiveness of the treatment was confirmed by plating the cells onto MHB-agar plate without dilution.

In-vivo evaluation of [^68^Ga]Ga-UBI complexes was carried out 24 h after induction of soft tissue infection and inflammation in wistar rats (*n* = 4). Tracer was prepared using the protocol mentioned in the radiolabeling section. To study *ex-vivo* biodistribution, ^68^Ga complexes were diluted in saline to achieve pH 5. ∼ 0.1 mL (~ 0.5 MBq, ~ 0.13 nmoles of peptides) of ^68^Ga complexes were injected in the rats via lateral tail vein. Animals were sacrificed at 1 h post-injection (p.i). The tissues and the organs were excised and the radioactivity associated with various tissues was counted in a flat type NaI (Tl) scintillation counter. The distribution of the activity in different organs was calculated as percentage of injected activity per gram of organ tissue (% ID/gm). To confirm focal nature of the infection and presence of bacteria at the target site, bacterial recovery was performed. Briefly, blood and thigh muscles bearing sterile inflammation (left) and infection (right) were weighed and homogenised using a tissue homogenizer. Subsequently, the samples were appropriately diluted and plated onto MHB-agar plates.

For PET imaging, ∼ 0.1 mL (∼ 11.1 MBq, ~ 1.8 nmoles of peptides) of radiotracer was injected into each rat (*n* = 3) through the lateral tail vein. Whole-body PET imaging was performed at 1 h p.i. using a time-of-flight PET scanner (Philips Gemini TF). A 3D iterative algorithm was used for image reconstruction. Paired t-test was utilized to estimate statistically significant differences among groups.

### Supplementary Information

**Additional file 1.** Data pertaining to the synthesis of a peptide-covalent probe, its purification via HPLC, and subsequent characterization using NMR and Mass spectrometry techniques.

## Data Availability

All data generated or analyzed during this study are included in this published article and in its Additional file 1.

## Abbreviations

APBA: 2-Acetylphenylboronic acid
AcCys-2-APBA: Acetyl-cysteine-2-acetyl boronic acid
PET: Positron emission tomography
SPECT: Single photon emission computed tomography
Lysyl-PG: Lysyl-phosphatidyl-glycerol
PG: Phosphatidyl-glycerol
NOTA: 1,4,7-Triazacyclononane-1,4,7-triacetic acid
UBI: Ubiquicidin
NODAGA: 1, 4, 7 Triazacyclononane 1-glutaric acid 4–7 acetic acid
AMR: Antimicrobial resistance
WHO: World Health Organization
MRI: Magnetic resonance imaging
CT: Computed tomography
AMP: Antimicrobial peptide
