# Supplementary material for: Expanding a peptide-covalent probe hybrid for PET imaging of S. aureus driven focal infections
Source: EJNMMI Radiopharm Chem. 2024 Mar 26;9:25. doi: 10.1186/s41181-024-00252-4 (PMC10965847; doi:10.1186/s41181-024-00252-4)
Supplement: Supplementary file 1 — Additional file 1. Data pertaining to the synthesis of a peptide-covalent probe, its purification via HPLC, and subsequent characterization using NMR and Mass spectrometry techniques. [file 41181_2024_252_MOESM1_ESM.doc]

**Electronic Supplementary Information**

**Expanding a Peptide-Covalent Probe Hybrid for PET Imaging of *S. aureus* Driven Focal Infections**

Jyotsna Bhatt Mitra^1,5^, Saurav Chatterjee^2^, Anuj Kumar^1^, Elina Khatoon^1^, Ashok Chandak^3^,

Sutapa Rakshit^4^, Anupam Bandyopadhyay^2^*, Archana Mukherjee^1,5^*

^1^Radiopharmaceuticals Division, Bhabha Atomic Research Centre (BARC), Mumbai, India

^2^Biomimetic Peptide Engineering Laboratory, Department of Chemistry,

Indian Institute of Technology, Ropar, Rupnagar, Punjab, India

^3^Board of Radiation & Isotope Technology, Navi Mumbai, India

^4^Radiation Medicine Centre, Parel, Mumbai, India

^5^Homi Bhabha National Institute, Anushaktinagar, Mumbai, India.

***Author for correspondence:**

Archana Mukherjee

Email: [archanas@barc.gov.in](mailto:archanas@barc.gov.in)

*ORCID ID :0000-0003-3068-1890*

Anupam Bandyopadhyay

Email: [anupamba@iitrpr.ac.in](mailto:anupamba@iitrpr.ac.in)

*ORCID ID:* [*0000-0002-3221-0315*](https://orcid.org/0000-0002-3221-0315)

1. **Materials**

1-(2,4-Dihydroxyphenyl) ethanone, *tert*-butyl bromoacetate, *N*-phenyl bis(trifluoromethanesulfonimide) (PhNTf_2_), 4-(dimethylamino) pyridine (DMAP), [1,1′-Bis(diphenylphosphino)ferrocene] dichloropalladium (II) (Pd(dppf)Cl_2_), potassium acetate and allylamine were purchased from GLR Innovations, India. Bis(pinacolato)diboron (B_2_pin_2_) was purchased from Avra, India. Potassium carbonate was purchased from LobaChemie, India. Dry DCM, Trifluoroacetic acid (TFA), *N, N*-Diisopropylethylamine (DiPEA) and Thioanisole were purchased from Spectrochem, India. Dry dioxane and N-methyl-2-pyrrolidone (NMP) were purchased from Finar, India. All the Fmoc-protected amino acids, (2-(1H-benzotriazol-1-yl)-1,1,3,3-tetramethyluronium hexafluorophosphate (HBTU) and Rink amide AM resin (loading capacity 0.67 mmol/g) were purchased from Chem-Impex Int’l Inc (Wood Dale, IL). 2,2-Dimethoxy-2-phenylacetophenone (DMPA) and 1,2-ethanedithiol were purchased from TCI Chemicals, India. Phenol was purchased from SRL, India. NODA-GA (^t^Bu)_3_ was purchased from CheMaTech, France. All reactions were monitored by thin-layer chromatography (TLC) on aluminium-backed silica gel 60 F_254_, 0.2 mm plates (Merck), and compounds were visualized under UV light (254 nm) or charred with appropriate solutions. Synthetic compounds were purified on 60-120 silica gel purchased from Finar unless otherwise specified.

1. **Instruments**

NMR data were recorded on a 400 MHz Jeol JNM ECS400 NMR spectrometer. Mass-spec data was generated by a Waters XEVO G2-XS QTOF mass spectrometer. Analytical HPLC and peptide purification were carried out on the Shimadzu Prominence UFLC system.

*Method for analytical RP-HPLC:* Waters Reliant C18 (5 µm, 4.6×250 mm) analytical column using mobile phase ACN-H_2_O (0.05% TFA) with a 1 mL/min flow rate. The gradient used: isocratic 10% ACN over 1 min, then a gradient from 10% to 50% ACN in 19 min, then a gradient from 50% to 90% ACN over 4 min, followed by isocratic 90% ACN over 2 min, then column equilibration at the initial isocratic gradient 10% ACN over 5 min.

*Method for semi-prep RP-HPLC:* Shimadzu shim-pack GIST C18 (5 µm, 10×250 mm) semi-preparative column was used with mobile phase ACN-H_2_O (0.05% TFA) at a flow rate 4 mL/min. The gradient used: isocratic 5% ACN over 2 min, then a gradient from 5% to 30% ACN over 26 min, then a gradient from 30% to 90% ACN over 3 min, then isocratic 90% ACN over 5 min, followed by column equilibration at the initial isocratic gradient 5% ACN over 5 min.

1. **Methods for chemical synthesis**

**Synthesis and characterization of UBI (29-41)-2-APBA^1^**

***Synthesis of 2-APBA alkene***

*Synthesis of* ***1***

2,4-Dihydroxybenzaldehyde (1.97 g, 13 mmol), *tert*-butyl bromoacetate (2.1 mL, 14.3 mmol, 1.1 eq.) and K_2_CO_3_ (1.8 g, 13 mmol, 1 eq.) were suspended in 26 mL acetone and refluxed at 65 °C for 5 hr. The reaction mixture was cooled and filtered to remove K_2_CO_3_. The organic layer was concentrated and filtered through silica gel to obtain 3.38 g (98%) white powder.

**^1^H NMR** (400 MHz, CDCl_3_): δ 7.81 (d, *J* = 8.8 Hz, 1H), 6.91 (dd, *J* = 8.8, 2.5 Hz, 1H), 6.79 (d, *J*= 2.4 Hz, 1H), 4.56 (s, 2H), 2.56 (s, 3H), 1.46 (s, 9H).

**^13^C NMR** (101 MHz, CDCl_3_): δ 202.8, 167.1, 165.1, 164.2, 132.6, 114.6, 107.9, 101.7, 83.0, 65.5, 28.1, 26.4.

**HRMS-ESI^+^**(*m/z*): Calc. [M+H] ^+^267.1232, Obs. 267.1229.

*Synthesis of* ***2***

**1** (3.3 g, 12.4 mmol), PhNTf_2_ (4.87 g, 13.6 mmol, 1.1 eq.), and DMAP (151.5 mg, 1.24 mmol, 0.1 eq.) were dissolved in 23 mL dry DCM and stirred at ice bath for 5 min. Et_3_N (3.5 mL, 24.8 mmol, 2 eq.) was added drop wise to the reaction mixture when the colour of the solution changed from light yellow to deep yellow. It was further stirred at room temperature for 4 hr, and then DCM was evaporated. The residue was dissolved in 300 mL EtOAc, washed with 120 mL 1N HCl, followed by 150 mL 10% Na_2_CO_3_ and the combined organic layers were washed with brine. Upon evaporation of the solvent, the product was purified through silica gel using 10% EtOAc/Hexane to obtain 3.54 g (72%) white solid.

**^1^H NMR** (400 MHz, CDCl_3_): δ 7.64 (d, *J* = 8.9 Hz, 1H), 6.48 (dd, *J* = 9.0, 2.5 Hz, 1H), 6.34 (d, *J* = 2.5 Hz, 1H), 4.53 (s, 2H), 2.55 (s, 3H), 1.48 (s, 9H).

**^13^C NMR** (101 MHz, CDCl_3_): δ 195.1, 166.6, 161.8, 148.4, 132.8, 124.9, 120.2, 117.1, 113.9, 109.7, 83.4, 65.9, 29.2, 28.0.

**HRMS-ESI^+^** (*m/z*): Calc. [M+H] ^+^399.0725, Obs. 399.0724.

*Synthesis of* ***3***

**2** (0.995 g, 2.5 mmol), Pd(dppf)Cl_2_ (73.2 mg, 0.1 mmol, 0.04 eq.), B_2_pin_2_ (0.953 g, 3.75 mmol, 1.5 eq.), and KOAc (612.5 mg, 6.25 mmol, 2.5 eq.) was taken together in a 25 mL oven-dried RB flask. 15 mL of dry dioxane was added, and the mixture was purged with argon for 20 min. The reaction mixture was then heated at 87 °C for 40 min with stirring. After completion of the reaction, as indicated by TLC, the reaction mixture was filtered through a pad of celite. Upon evaporation of the solvent, the residue was purified through silica gel using 12% EtOAc/Hexane to obtain a 670mg (76%) yellowish solid.

**^1^H NMR** (400 MHz, CDCl_3_): δ 7.76 (d, *J* = 8.6 Hz, 1H), 6.92 (d, *J* = 2.6 Hz, 1H), 6.87 (dd, *J* = 8.7, 2.7 Hz, 1H), 4.55 (s, 2H), 2.54 (s, 3H), 1.46 (s, 9H), 1.41 (s, 12H).

**^13^C NMR** (101 MHz, CDCl_3_): δ 198.6, 167.4, 161.5, 134.3, 130.7, 117.8, 114.7, 83.7, 83.2, 82.9, 77.4, 77.1, 76.8, 65.7, 28.1, 25.0, 24.6.

**HRMS-ESI^+^**(*m/z*): Calc. [M-pin-OH] ^+^ 277.1247, Obs. 277.1239.

*Synthesis of* ***2-APBA alkene***

670 mg of **3** was dissolved in 3 mL cold neat TFA, forming a yellow-coloured solution. It was then stirred at room temperature for 15 minutes. TLC analysis indicated the completion of the reaction. Residual TFA was evaporated on a rotary evaporator 3 times with 50 mL DCM, and 216 mg of yellow solid was obtained by precipitation using 10% EtOAc/Hexane, which was used for the next step without further purification.

216 mg of the acid derivative (0.91 mmol) and HBTU (345 mg, 0.91 mmol, 1 eq.) were dissolved in 1 mL DMF and stirred at room temperature. To this solution, DiPEA (232 µL, 1.36 mmol, 1.5 eq.) and allylamine (75 µL, 1 mmol, 1.1 eq.) were added with a 2 min interval in stirring condition. Reaction completion after 40 min was indicated by TLC analysis. The reaction mixture was diluted in 200 mL EtOAc, and the organic layer was washed with 75 mL chilled 1N HCl, 100 mL chilled 5% NaHCO_3_, and 60 mL brine. After evaporation of the organic layer, the residue was purified through silica gel using 40% EtOAc/Hexane to obtain 245 mg (75%) yellowish gummy solid.

**^1^H NMR** (400 MHz, 5% CD_3_OD/CDCl_3_): δ 7.81 (d, *J* = 8.6 Hz, 1H), 7.01 (d, *J* = 2.6 Hz, 1H), 6.89 (dd, *J* = 8.5, 2.6 Hz, 1H), 6.59 (brs, 1H), 5.89-5.79 (m, 1H), 5.23 – 5.14 (m, 2H), 4.57 (s, 2H), 3.99 – 3.96 (m, 2H), 2.56 (s, 3H), 1.43 (s, 12H).

**^13^C NMR** (101 MHz, 5% CD_3_OD/CDCl_3_): δ 168.0, 163.2, 160.7, 134.5, 133.3, 131.1, 118.5, 116.8, 113.9, 84.0, 66.9, 38.5, 24.8.

**HRMS-ESI^+^** (*m/z*): Calc. [M-pin-OH] ^+^ 260.1094, Obs. 260.1104.

***Synthesis of AcCys-APBA***

AcCys-OH (220 mM, 1.1 eq), APBA (200 mM, 1eq), and DMPA (20 mM) were dissolved in NMP (1% TFA) and stirred for 20 min in the presence of hν ~365 nm light source in a dark place. The reaction mixture was diluted to 1 mL with 1 N HCl and washed with 2 mL EtOAc. The crude residue was purified through RP-HPLC, and the NMR data for the same are presented.

*Method for analytical HPLC* (Shimadzu Prominence UFLC): Waters Sunfire C18 (5 µm, 4.6×250 mm) analytical column was used with mobile phase ACN-H_2_O (0.05% TFA) at a flow rate of 1 mL/min. The gradient used: isocratic 10% ACN for 1 min, then a gradient from 10% to 90% ACN in 19 min, then isocratic 90% ACN for a min, then column equilibration at an initial isocratic gradient 10% ACN over 4 min.

*ESI-MS analysis:* Calc. [M-pin-OH] ^+^423.14, Obs. 423.13

*Analytical RP-HPLC: R_t_* = 6.99 min

***Synthesis of UBI-APBA***

Peptide synthesis was carried out by standard SPPS Fmoc-deprotection strategy on Rink amide resin. Briefly, three equivalents of the commercially available amino acids, HBTU as a coupling agent and DiPEA as a base, were used for the coupling reaction for 15 min. Fmoc deprotection was achieved using 3 mL 20% piperidine/DMF twice for 3 min each, followed by washing with 3 mL DMF six times. The peptides were cleaved off the resin and globally deprotected with reagent K (82.5% TFA, 5% H_2_O, 2.5% EDT, 5% Thioanisole and 5% phenol). Precipitation with chilled diethyl ether gave the crude peptides, which were purified by RP-HPLC. The mass and purity of the peptide were determined by using LC-MS (Waters) and HPLC (Shimadzu) to be >95%.

*ESI-MS analysis:* Calc. [M+3H]^3+^ 618.40, Obs. 618.01

*Analytical RP-HPLC: R_t_* = 2.1 min

***Installation of 2-APBA alkene on UBI (29-41)***

2-APBA alkene was installed on the C-terminal Cys residue of UBI by thiol-ene click reaction. 10 mM peptide, 12 mM 2-APBA alkene and 8 mM DMPA were dissolved in 200 µL1:1 NMP: H2O (1% TFA) in a 1.5 mL clear glass vial. It was kept in the centre between two Philips TL 8W BLB light sources, 7 cm apart with λ~365 nm for 20 min. The reaction mixture was then diluted with 800 µL 20% ACN/H_2_O (0.05% TFA), filtered through a 0.22 µm nylon filter and subjected to purification by RP-HPLC.

*ESI-MS analysis:* Calc. [M+4H]^4+^ 533.03, Obs. 533.01

*Analytical RP-HPLC: R_t_* = 2.2 min


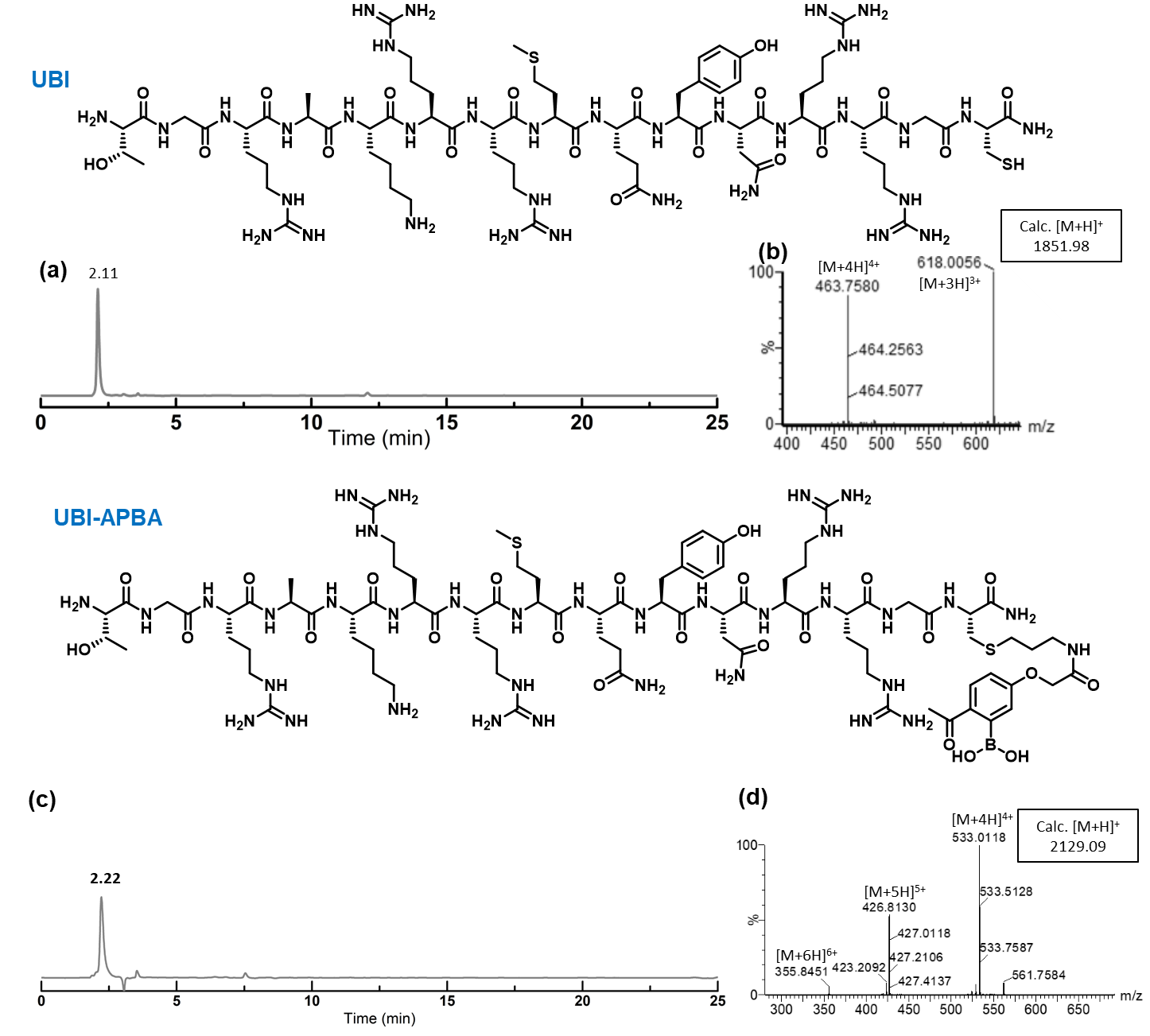


**Fig. S1:** UBI peptide data: a) HPLC purity at 220 nm; b) Mass data; UBI-APBA peptide data: c) HPLC chromatogram at 220 nm showing>95% purity of the product; d) Mass datum confirms the product’s identity.

***Synthesis of NODAGA-UBI (29-41)-2-APBA***

Peptide synthesis was carried out by standard SPPS Fmoc-deprotection strategy on Rink amide resin. Briefly, three equivalents of the commercially available amino acids, HBTU as a coupling agent and DiPEA as a base, were used for the coupling reaction for 15 min. Fmoc deprotection was achieved using 3 mL 20% piperidine/DMF twice for 3 min each, followed by washing with 3 mL DMF six times. NODAGA (^t^Bu)_3_was coupled at the N-terminal. The peptides were cleaved off the resin and globally deprotected with reagent K (82.5% TFA, 5% H_2_O, 2.5% EDT, 5% Thioanisole and 5% phenol) at 50 ºC (to cleave all ^t^Bu groups from NODA-GA) for 3 hr. Precipitation with chilled diethyl ether gave the crude peptides, which were purified by RP-HPLC. The mass and purity of the peptide were determined by using LC-MS (Waters) and HPLC (Shimadzu) to be 95%, as shown in the main text fig. 2. 2-APBA were installed on NODAGA-UBI following the same procedure.

*ESI-MS analysis:* Calc. [M+5H]^5+^498.25, Obs. 498.21

*Analytical RP-HPLC: R_t_* = 2.4 min


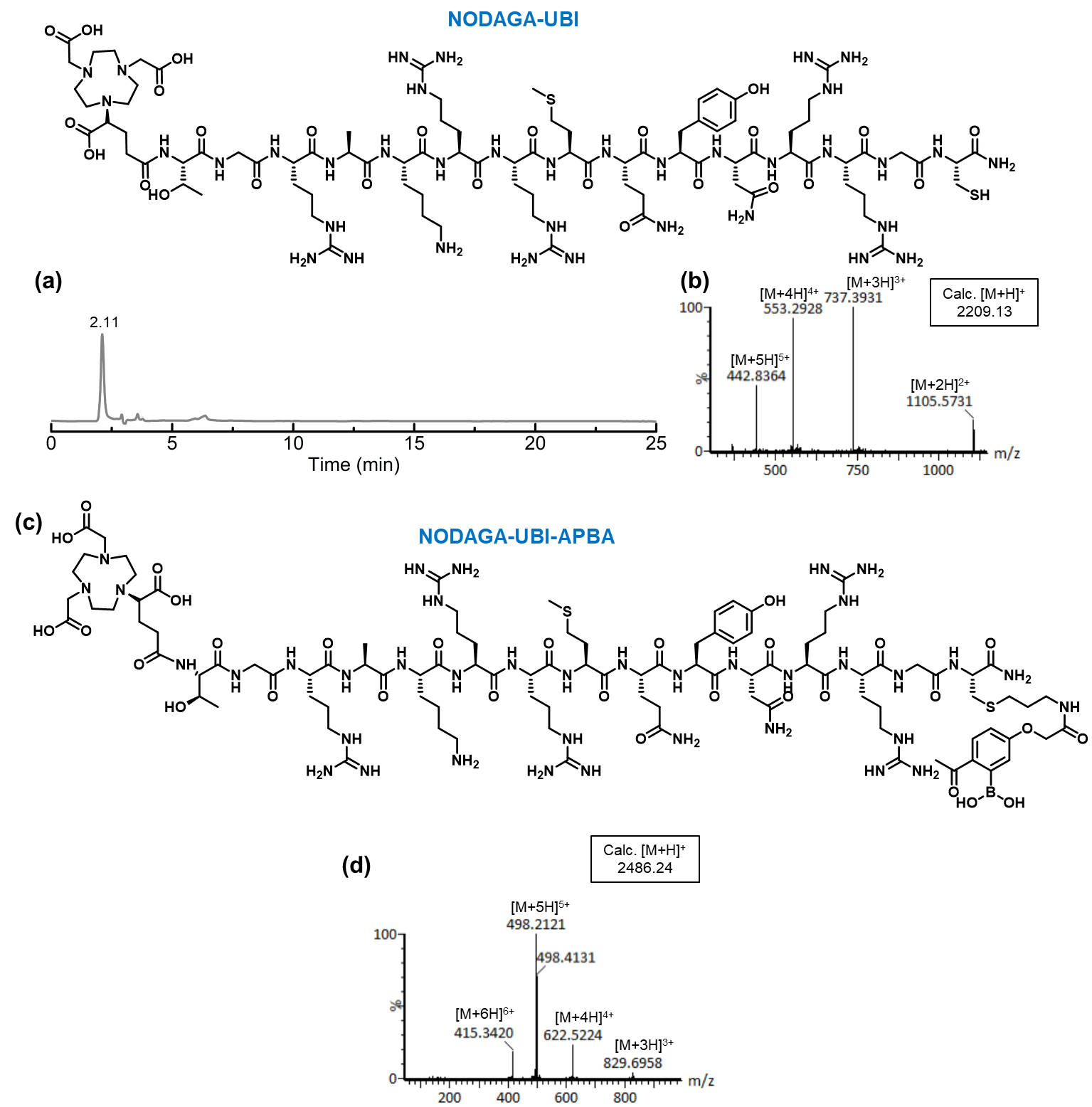


**Fig S2:** Chemical structure of NODAGA-UBI: a) HPLC chromatogram determines purity at 220 nm; b) Mass datum confirms peptide’s identity. (c) Chemical structure of NODAGA-UBI-APBA and its identity in d) mass spectrometry. Purity of NODAGA-UBI-APBA is shown in the main text fig. 2.

1. **NMR Data**


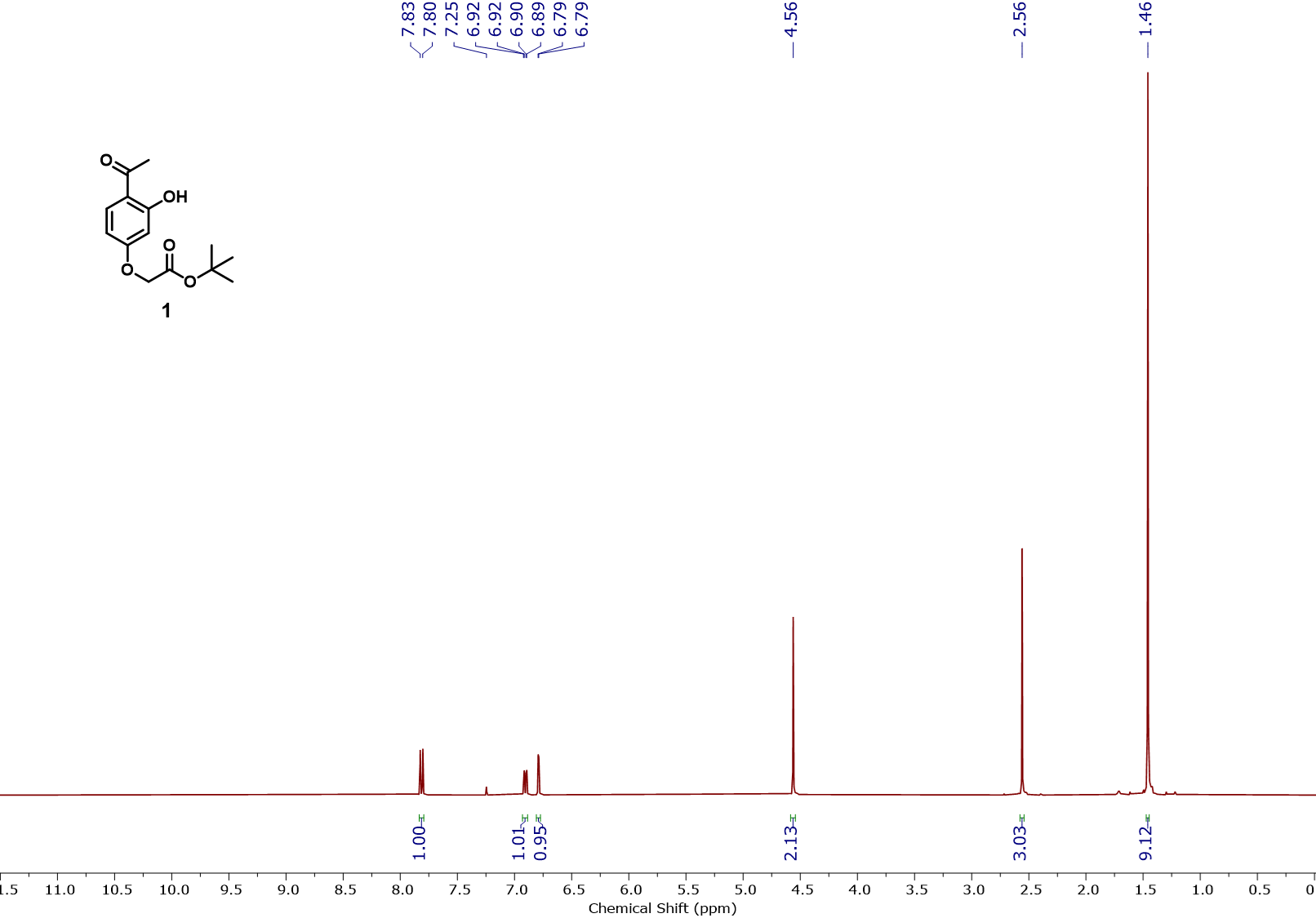


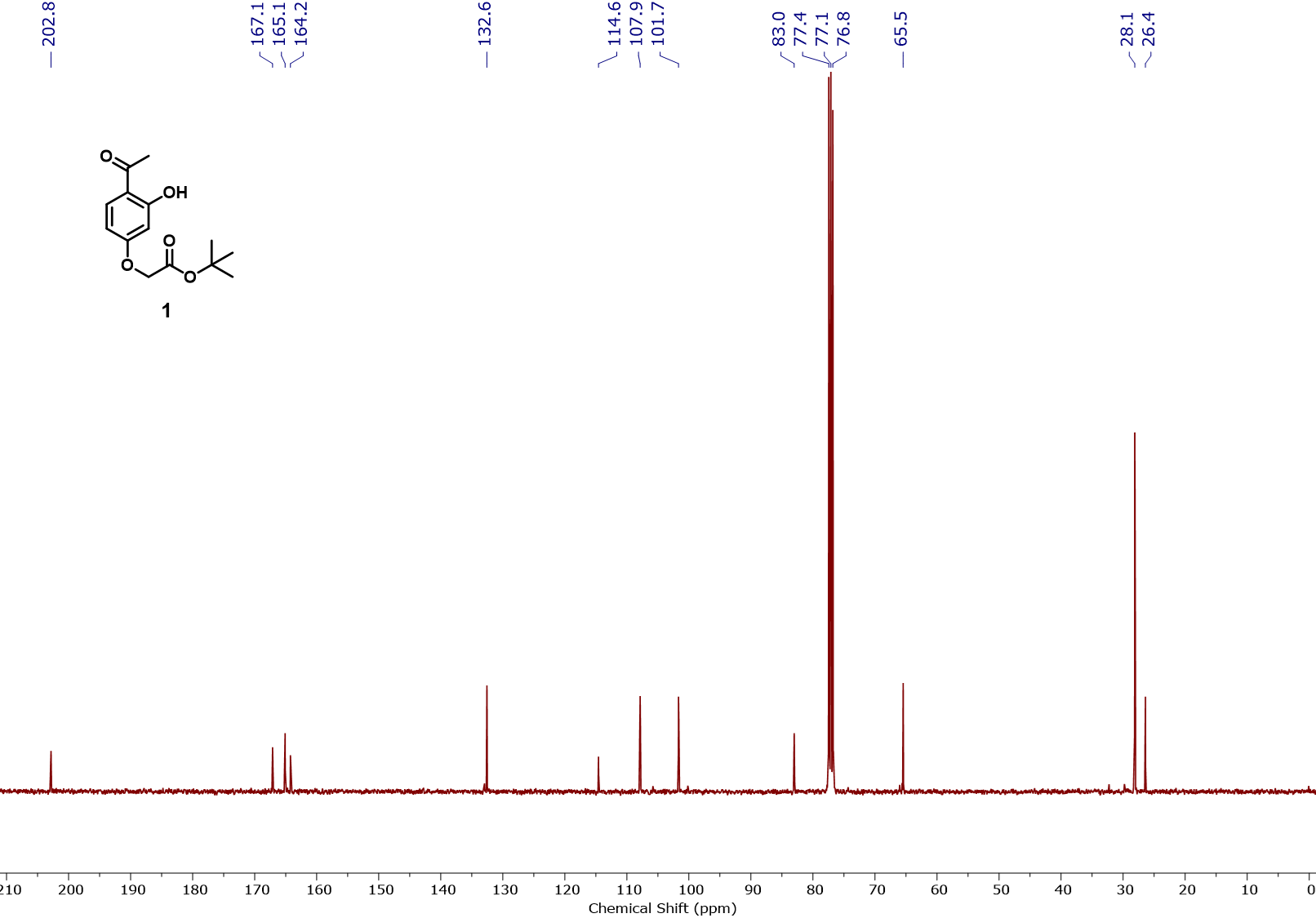


**Fig. S3:**^1^H and ^13^C NMR spectra of compound 1 in CDCl_3_.


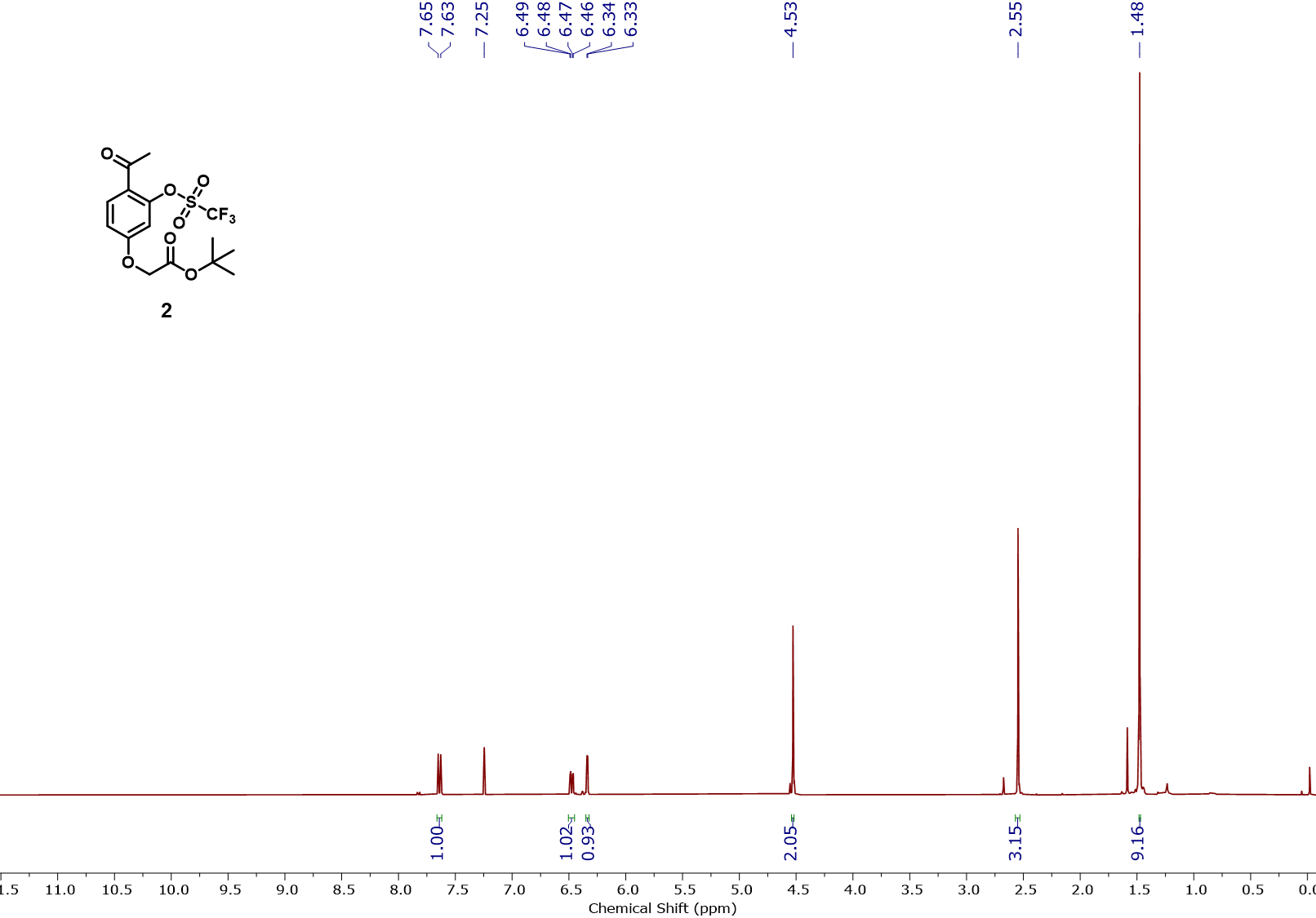


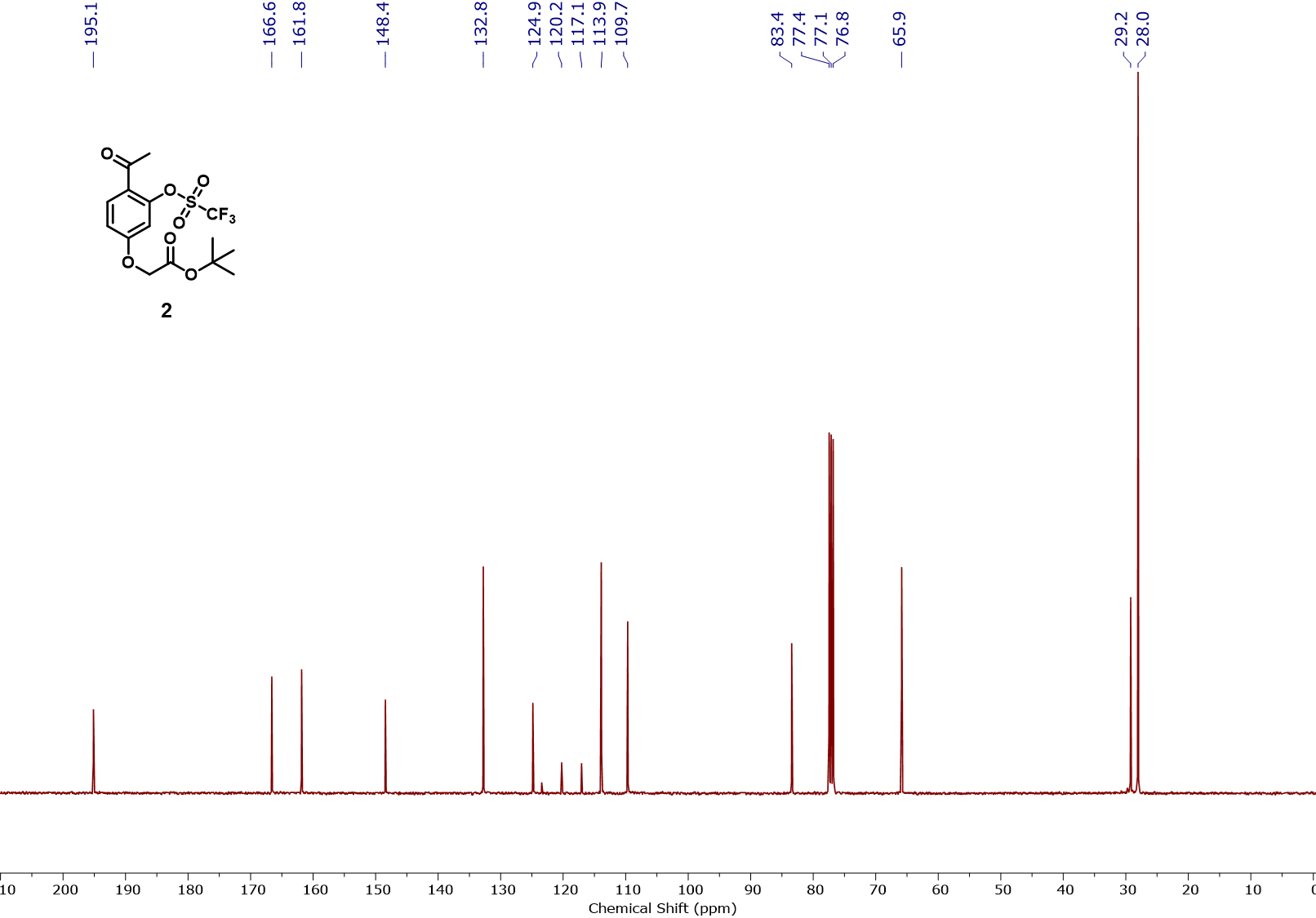


**Fig. S4:**^1^H and 1^3^C NMR spectra of compound 2 in CDCl_3_.


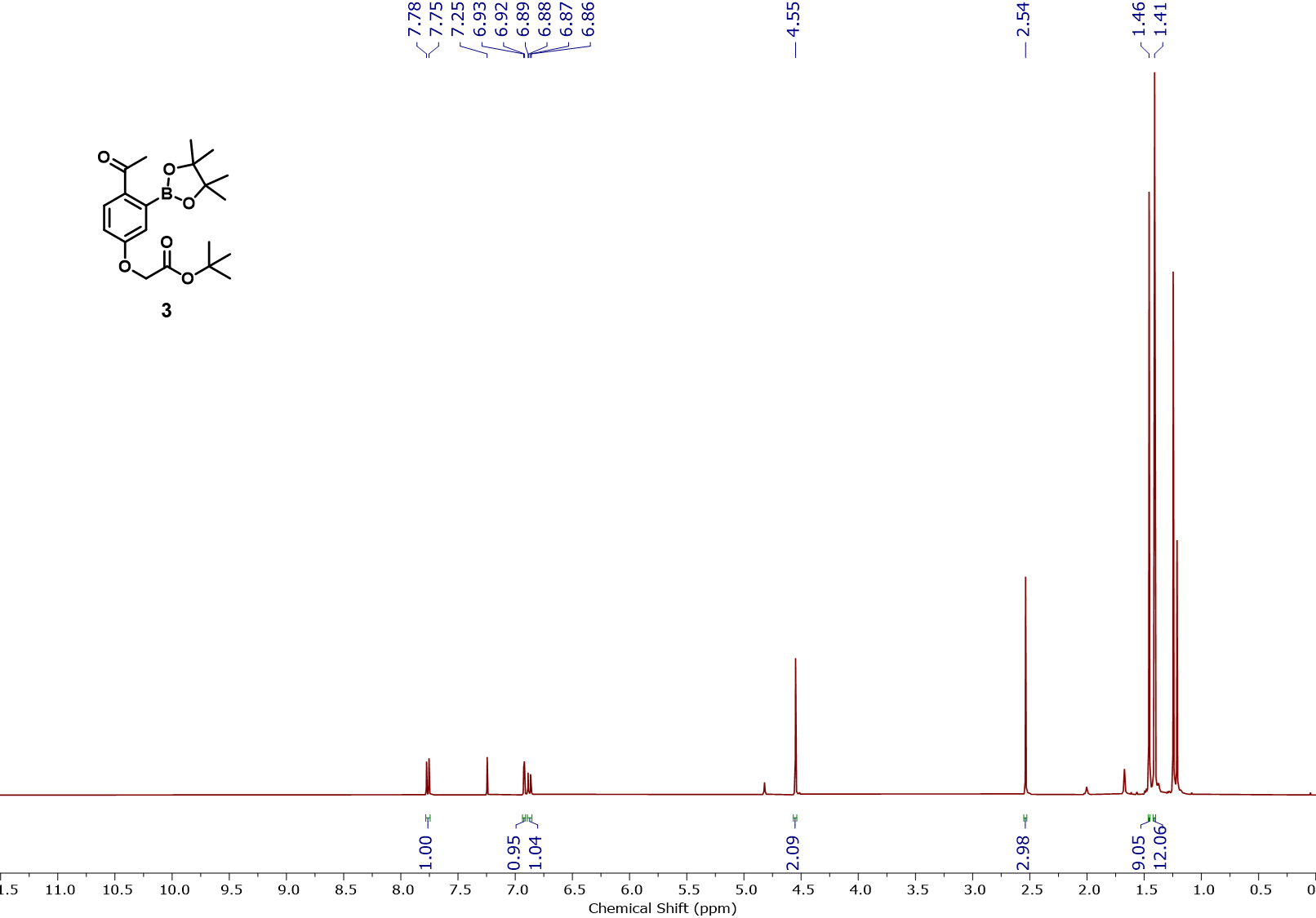


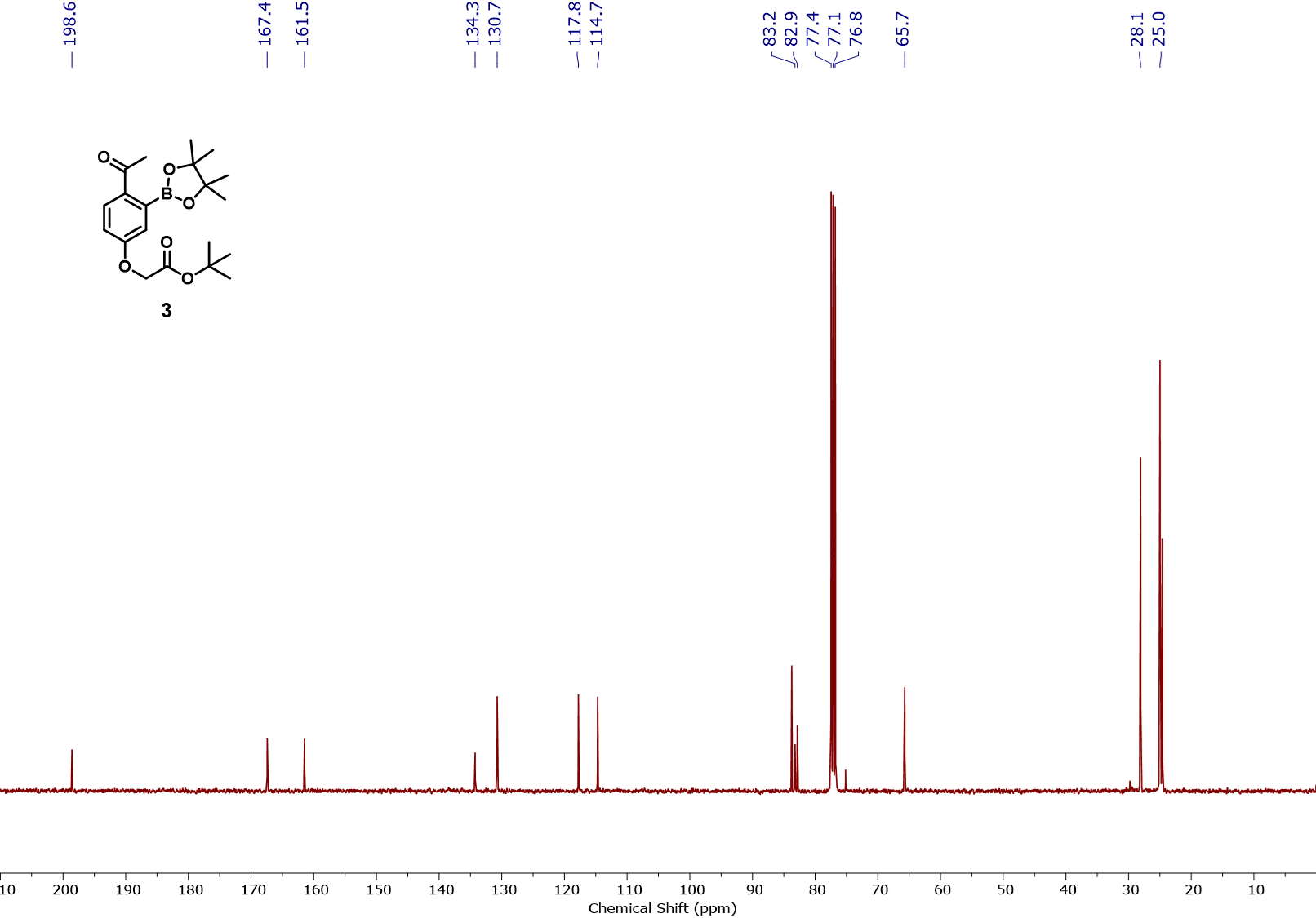


**Fig. S5:**^1^H and ^13^C NMR spectra of compound 3 in CDCl_3_.


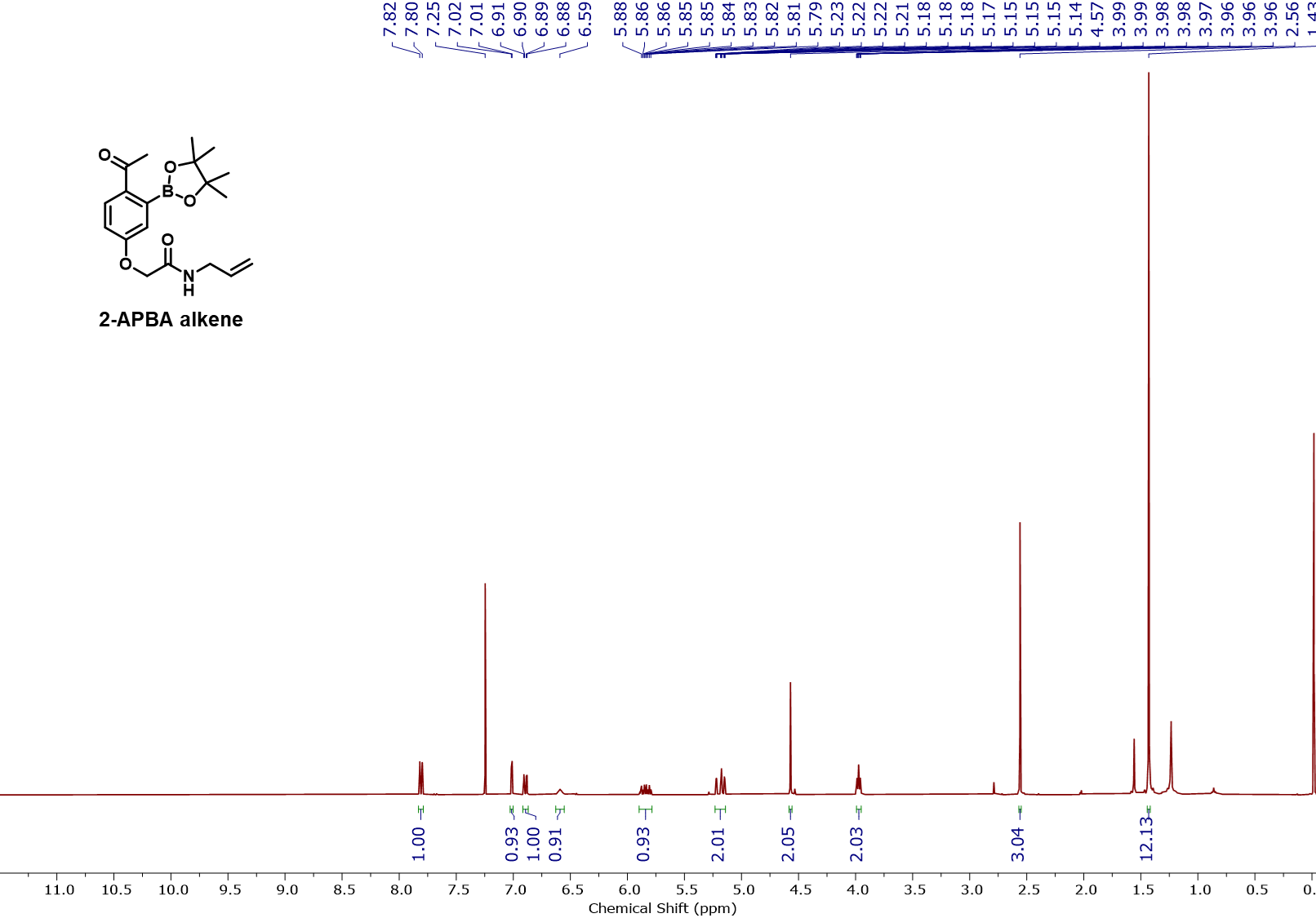


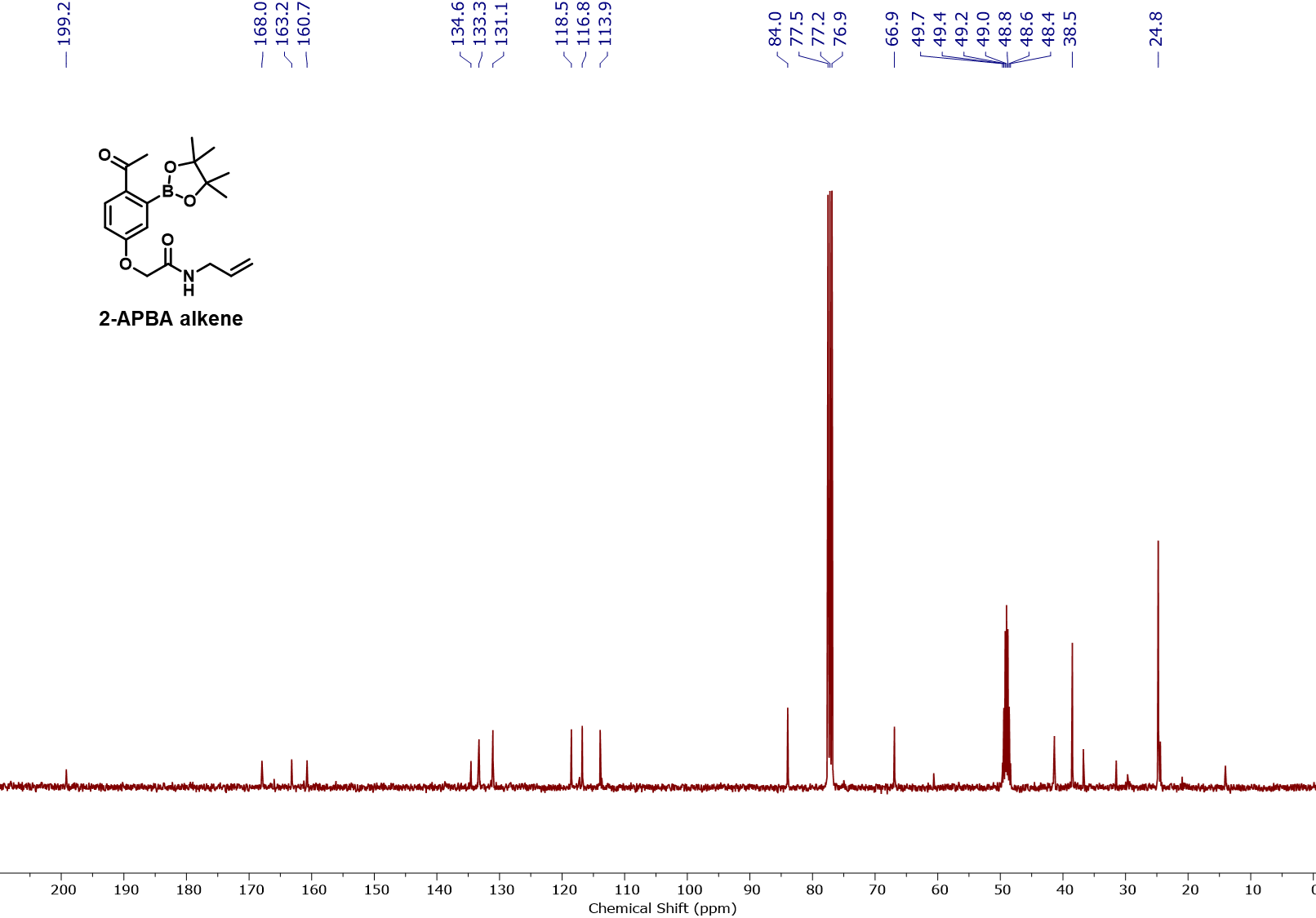


**Fig. S6:**^1^H and ^13^C NMR spectra of compound 2-APBA alkene in 5% CD_3_OD/CDCl_3_.


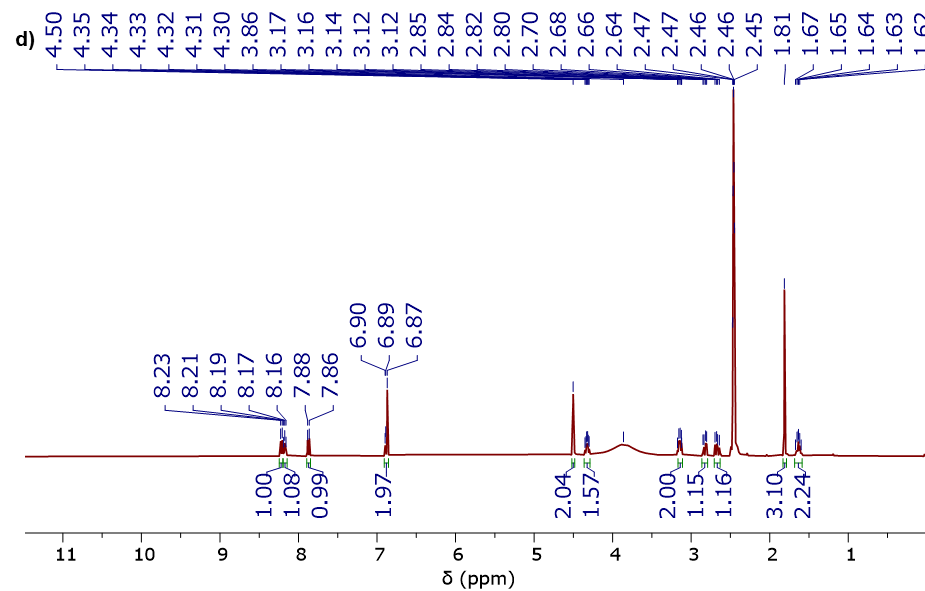


**Fig. S7:** a) NMR spectrum of pure AcCys-2-APBA in DMSO-*d*_6_.

**References**

1. Bhatt Mitra J, Chatterjee S, Kumar A, Bandyopadhyay A, Mukherjee A. Integrating a covalent probe with ubiquicidin fragment enables effective bacterial infection imaging. RSC Med Chem. 2022;13(10):1239-45.
